# Neural Network-Based LoRa Received Signal Strength Indicator Fingerprint Identification for Indoor Localization of Mobile Robots

**DOI:** 10.3390/s26072127

**Published:** 2026-03-30

**Authors:** Chandan Barai, Meem Sarkar, Ushnish Sarkar, Subhabrata Mazumder, Abhijit Chandra, Tapas Samanta, Hemendra Kumar Pandey

**Affiliations:** 1 Homi Bhabha National Institute, 2nd Floor, BARC Training School Complex, Anushaktinagar, Mumbai 400094, Maharashtra, India; u.sarkar@vecc.gov.in (U.S.); tsamanta@vecc.gov.in (T.S.); hkpandey@vecc.gov.in (H.K.P.); 2 Variable Energy Cyclotron Centre, 1/AF, Canal Side Rd, AF Block, Sector 1, Bidhannagar, Kolkata 700064, West Bengal, India; subhabrata@vecc.gov.in; 3 Indian Institute of Engineering Science and Technology, Botanical Garden Area, Howrah 711103, West Bengal, India; meemsarkar@gmail.com; 4 Department of Instrumentation & Electronics Engineering, Jadavpur University, Kolkata 700106, West Bengal, India; abhijit.chandra@jadavpuruniversity.in

**Keywords:** RSSI fingerprint, LoRa, time series RSSI preprocessing, SSIM, entropy of fingerprint database, MLP

## Abstract

This paper presents an indoor self-localization framework for mobile robots, an essential component for automation in Industry 4.0 and smart environments. We evaluate a Received Signal Strength Indicator (RSSI) fingerprinting technique utilizing Long-Range (LoRa) technology to overcome the challenges of congested indoor settings. To optimize communication parameters, the Structural Similarity Index Measure (SSIM) was employed to select the most effective spreading factor, while the entropy of the RSSI database was calculated to verify fingerprint stability. For positional prediction, a Multi-layer Perceptron (MLP) neural network was developed to classify the location of the target within a grid-based experimental setup, featuring cells spaced 60 cm apart. The MLP achieved a validation accuracy of 91.8 percent during training and demonstrated high precision in classifying grid regions within a signal-dense environment. For scenarios where slow-moving robots (5 cm/s) are required, like radiation mapping, this method provide highly accurate high-level localization data.These results suggest that the proposed LoRa-MLP integration provides a robust, low-power solution for high-accuracy indoor positioning systems (IPSs) in modern industrial infrastructure.

## 1. Introduction

In recent years, Location-Based Services (LBSs) have become pivotal across sectors including healthcare monitoring, emergency and disaster management, security surveillance, and warehouse logistics. The bloom of robotics and transition towards Industry 4.0, smart homes, smart offices and intelligent buildings depend critically on LBS infrastructure [1]. Although Global Navigation Satellite Systems (GNSSs)—such as the Global Positioning System (GPS) and Assisted Global Positioning System (AGPS)—provide reliable information in outdoor positioning, they prove ineffective in the case of indoor navigation. This failure is due to severe signal attenuation from multipath effects, deep shadowing, reflections, and structural obstructions [2,3]. Moreover, GNSSs are accurate to within a few meters, and they rarely achieve sub-meter precision, which is essential for indoor localization.

Autonomous indoor mobile robots serve as a major element of the current industrial structure. Demands for automation that exceed human speed and accuracy, or situations where human safety is concerned, are met by employing autonomous robots. Indoor mobile robots produce substantial socio-economic benefits in laboratories, logistics, healthcare, and transportation [4]. Autonomous navigation, however, requires accurate self-localization capabilities [5]. Conventional wheel-based encoders are prone to accumulating odometry errors over time, which ultimately compromises position estimates [6,7]. As a consequence, external Indoor Positioning Systems (IPSs) remain important for precise spatial information. Existing literature documents present various IPS methodologies, like Inertial Measurement Units (IMUs), which employ dead reckoning by fusing magnetometer, accelerometer, and gyroscope data [8]. IMUs are resistant to line-of-sight (LoS) obstructions, but they heavily drift on uneven surfaces or in dynamic environments [9]; hence, they serve primarily as supplementary methods [10]. Visible Light Communication (VLC) via light-emitting diodes (LEDs) exhibits high accuracy and electromagnetic immunity but requires LoS. It is sensitive to ambient lighting conditions and demands substantial power [11]. IPS methods using ultrasonic and infrared-based sensors offer precision within limited ranges (6–10 ft) but suffer from noise susceptibility. Furthermore, complex calibrations are required [12,13,14]. Computer vision localization is very accurate, yet it relies heavily on consistent, high-intensity illumination and ambient light [9,10].

### Related Works in Received Signal Strength Indicator (RSSI)-Based Indoor Positioning

RF technologies dominate indoor positioning systems due to their reliability and cost-effectiveness. Most of the systems use either triangulation or fingerprinting [3]. Triangulation methods based on Time of Arrival (ToA), Angle of Arrival (AoA), or Phase of Arrival (PoA) demand precise hardware synchronization and a wide bandwidth. Hence, they require a complicated system structure [15]. Fingerprinting, on the other hand, matches real-time RSSI data against a database of signal signatures. The systems are highly accurate, less complicated and compatible with highly accessible protocols like Wi-Fi, Bluetooth Low Energy (BLE), ZigBee, or Long Range (LoRa) [9].

Recent advancements, such as WiMap [16], have shown the efficacy of high-fidelity wireless signal mapping in order to boost sensing performance. WiMap focuses on spatial distribution accuracy. The reported work utilizes a grid-based classification. In the reported work, by segmenting the environment into multiple grids, we mitigate the impact of the occasional outliers (reaching up to 5.43 m) observed in our Cumulative Distribution Function (CDF) analysis and ensure that the majority of localization events lie at a minimum error threshold. This stability is particularly relevant when compared to conventional signal distribution modeling [17].

Received Signal Strength (RSS) measurements have profoundly influenced IPS development. A landmark study in 2005 introduced RSS-based tracking via IEEE 802.11 Access Points (APs) [18] in zero-configuration settings [19]. The study also pointed Received Signal Strength Indicator (RSSI) sensitivity to temperature, humidity, and human-induced movements in the environment.

Current research focuses on robust long-range solutions. Islam et al. [20] reported LoRa RSSI’s superior stability relative to Wi-Fi and BLE in congested environments. Even in signal-dense areas, Long-Range Wide-Area Network (LoRaWAN) maintains effective coverage [21].

Implementation of Machine Learning (ML) techniques has remarkably enhanced localization precision. Machine learning-based position prediction works well with LoRa signals [22]. LoRa demonstrates resilience to multipath interference and signal loss with optimized radio parameters [23]. Wang et al.’s deep learning architecture (DeepFi algorithm) significantly reduced localization errors compared to previous conventional methods [24]. More recent works have explored the synergy between deep learning and LoRa RSSI to produce precise indoor localization [25]. To make fingerprinting collection less tedious, researchers have introduced dynamic systems that use mobile robots to update fingerprint databases autonomously, keeping IPS effective over a longer time [26]. Table 1 summarizes these key advances.

Despite many available techniques, a universal solution is not provided by any single system, as there are always trade-offs between accuracy, cost of the system, power consumption, and scalability. This work aims to build an Radio Frequency (RF)-based IPS tailored for a mobile robot collecting radiation measurements in laboratory settings. We focus on the applicability of RSSI fingerprinting using LoRa technology as the enabling communication layer. Unlike the existing body of literature, the reported work introduces a distinct approach that connects raw signal data to reliable spatial classification. While earlier foundational studies have established the stability of LoRa over Wi-Fi and BLE [20] and have applied deep learning techniques for the reduction of error [24], this study differentiates itself by introducing a distinct preprocessing and optimization layer.

In addition to the dual-metric verification of the fingerprint database, there are a few additional contributions that make up the novel aspects of this project: first, the use of the Structural Similarity Index Measure (SSIM) to identify the optimal spreading factor to be used for LoRa communication. The second contribution is the use of Shannon entropy to measure the stability of the RSSI signature data prior to the construction of the fingerprint map via network training. Unlike previous works, where broader coverage but with lower resolution was reported [21] or adaptive collection methods are implemented [26], this research uses high-resolution (60 cm granularity) grid-based classification of the cells in the fingerprint map, as opposed to a lower-resolution classification. By using a multi-layer perceptron (MLP) network, the classification of each cell within the map is found with an accuracy of 91.8% in a congested laboratory environment. The proposed LoRa RSSI fingerprinting scheme was demonstrated and experimentally validated on actual physical hardware instead of being simulated in software. The optimum LoRa spreading factor was selected from multiple configurations based upon the maximum value of the structural similarity (SSIM) between the fingerprint maps collected at different LoRa spreading factors. The optimum LoRa antenna height was similarly selected from multiple configurations, based upon the minimum value of the Shannon entropy of the RSSI signatures collected at different LoRa antenna heights. Collectively, the selection of these parameters, along with a dedicated preprocessing pipeline to generate the fingerprint map data, resulted in a very stable fingerprint map dataset, which enabled a shallow MLP classifier to accurately determine the location of the grid cells. Hence, the architecture remained computationally lightweight enough to be deployed on embedded edge computing platforms.

This research has been divided into four sections. The Section 2 describes the theoretical foundations of RSSI fingerprinting and LoRa technology. The Section 3 describes the system architecture and experimental setup. The Section 4 describes the experimental results. The Section 5 and Section 6 presents the conclusions. 

## 2. LoRa RSSI Fingerprint-Based IPS

RSSI is the measurement of the received signal strength indicator at various locations within the arena. Across several known positions called reference points (RPs), measurements capture how strong signals appear in practice. Each wireless access point (AP) contributes data that gets logged separately over time. As demonstrated in [27], multiple factors influence the positioning of APs and quantity of RPs inside an experimental arena. At any location within the arena, the RSSI signature comprises RSSI values from distinct APs. An RSSI fingerprint encompasses multiple signatures acquired at various points across the localization region. The experimental area is divided into reference points (RPs), $P=p_{j}=\left(x_{j},y_{j}\right),|,j=1,\ldots ,N$, where *P* represents the set of RPs in real-world Cartesian coordinates. The RSSI signature data is recorded and stored at discrete time intervals $t_{m}$, m=1,…,M, providing RSSI values ${r_{i}}^{j}\left(t_{1}\right)\ldots {r_{i}}^{j}\left(t_{m}\right)$ at each RP, where *i* indexes the APs from the set $\xi =AP^{1},\ldots AP^{L}$. The fingerprint is created by collecting an identical number of training samples, *M*, per RP. RSSI fingerprints from all APs at time $t_{m}$ and location $p_{j}$ are assembled into the vector $r_{j}\left(t_{m}\right)={\left[{r_{j}}^{1}\left(t_{m}\right),\ldots {r_{j}}^{L}\left(t_{m}\right)\right]}^{T}$.

### 2.1. RSSI Fingerprint-Based IPS

The fingerprint, representing the complete radio map at time interval $t_{m}$, is formulated in [27] as follows:

(1) $$\begin{array}{cc} R(t_{m}) & =(r_{1}\left(t_{m}\right),\ldots ,r_{N}\left(t_{m}\right)) \end{array}$$

(2) $$\begin{array}{cc} & \;=\left[\begin{array}{cccc} r_{1}^{1}\left(t_{m}\right) & r_{2}^{1}\left(t_{m}\right) & \ldots & r_{N}^{1}\left(t_{m}\right)\\ r_{1}^{2}\left(t_{m}\right) & r_{2}^{2}\left(t_{m}\right) & \ldots & r_{N}^{2}\left(t_{m}\right)\\ \vdots & \vdots & \ddots & \vdots \\ r_{1}^{L}\left(t_{m}\right) & r_{2}^{L}\left(t_{m}\right) & \ldots & r_{N}^{L}\left(t_{m}\right) \end{array}\right],\;m=1,\ldots ,M \end{array}$$

Furthermore, the vectors are defined as ${r_{j}}^{i}={\left[{r_{j}}^{i}\left(t_{1}\right)\ldots {r_{j}}^{i}\left(t_{M}\right)\right]}^{T}$, $r^{i}\left(t_{m}\right)={\left[{r_{1}}^{i}\left(t_{m}\right)\ldots {r_{N}}^{i}\left(t_{M}\right)\right]}^{T}$, and $r_{j}\left(t_{m}\right)={\left[{r_{j}}^{1}\left(t_{m}\right)\ldots {r_{j}}^{L}\left(t_{M}\right)\right]}^{T}$, denoting RSSI signatures across time instants, RPs, and APs, respectively. For RSSI fingerprints recorded over sequential time instances $R(t_{m})$, temporal averaging yields the time-averaged radio map, denoted in [27] as

(3) $$\psi =\left(\psi_{1},\ldots ,\psi_{N}\right)=\left[\begin{array}{ccccccc} \psi_{1}^{1} & \ldots & \psi_{N}^{1}\;\vdots & \ddots & \vdots \;\psi_{1}^{L} & \ldots & \psi_{N}^{L} \end{array}\right]$$

In the present study, RSSI fingerprints were acquired via a modified protocol incorporating averaging alongside supplementary preprocessing, as detailed in the following section. Upon offline fingerprint collection, a mobile user device captures the RSSI signature $y={\left(y^{1}\ldots y^{L}\right)}^{T}$ at an unknown location during the online phase. The user position $p=(\ddot{x},\ddot{y})$ is then estimated using the positioning algorithm. Fingerprint-based localization algorithms are broadly categorized into deterministic [28] and probabilistic variants [29,30,31]. Deterministic methods estimate position by identifying RPs whose RSSI signatures most closely match the online-phase observation:

(4) $$p^{\prime }=\arg\min_{j=1,\ldots ,K}d\left({\tilde{r}}_{j},y\right)$$

Here, ${\tilde{r}}_{j}$ signifies the signature at the *j*th RP, and $d({\tilde{r}}_{j},y)$ denotes a distance metric [32]. When employing time-averaged signatures, $\Psi_{j}$ substitutes for ${\tilde{r}}_{j}$. The nearest fingerprint’s signal space is designated as the target’s position [33]. Common metrics include cosine similarity [34], Euclidean distance [35,36,37], and Tanimoto similarity [38]. Deterministic approaches offer computational simplicity and ease of implementation. *K*-nearest neighbors (K-NN) exemplifies low-complexity deterministic methods, whereas advanced techniques such as linear discriminant analysis (LDA) [39] and support vector machines (SVM) [40] demand greater resources yet yield superior accuracy. A canonical metric, Euclidean distance, is expressed as

(5) $$d\left({\tilde{r}}_{j},y\right)={∥y-{\tilde{r}}_{j}∥}_{2},\;j=1,\ldots ,K$$

The weighted K-NN (wK-NN) algorithm computes position as a weighted coordinate sum [41]:

$$\begin{array}{c} W_{k}=\frac{1}{{∥y-\tilde{r}j∥}_{2}}/\sum {j=1}^{k}{∥y-\tilde{r}j∥}_{2} \end{array}$$

(6) $$p^{\prime }\mathrm{wK}-\mathrm{NN}=\sum_{k}p^{\prime }W_{k}$$

Deterministic approaches fall short when it comes to tracking the evolving nature of indoor wireless signals. For this reason, most current RSSI fingerprint-based indoor localization systems rely on probabilistic algorithms. These methods incorporate spatiotemporal dynamics, collaborative localization, and even motion cues [27,42]. By using statistical inference, they continuously match real-time measurements with stored fingerprints [43] and also model signal behavior. Collecting fingerprints over time allows these systems to create a statistical model of the wireless channel.

Central to these probabilistic techniques is maximum a posteriori (MAP) estimation [38,44]. The objective is to determine the most probable user position given the observed RSSI vector, mathematically expressed as

(7) $$p^{\prime }=\arg\max_{j=1,\ldots ,N}f\left(\frac{p_{j}}{y}\right)$$

where $f(p_{j}|y)$ represents the probability that the user is located at $p_{j}$ given the measurement y. Using Bayes’ theorem, this can be rewritten as

(8) $$f\left(\frac{p_{j}}{y}\right)=\frac{f(p_{j},y)}{f(y)}=\frac{f\left(\frac{y}{p_{j}}\right)f\left(p_{j}\right)}{\sum_{j=1}^{N}f\left(\frac{y}{p_{j}}\right)f\left(p_{j}\right)}$$

If there is no prior information about the user’s location ($f\left(p_{j}\right)=\frac{1}{N}$), the denominator serves to normalize the probabilities, and $f(p_{j})$ is a constant. With this uniform prior, the expression reduces to maximum likelihood estimation (MLE) [45]:

(9) $$p^{\prime }=\arg\max_{j=1,\ldots ,N}f\left(\frac{y}{p_{j}}\right)$$

**Figure 1:**
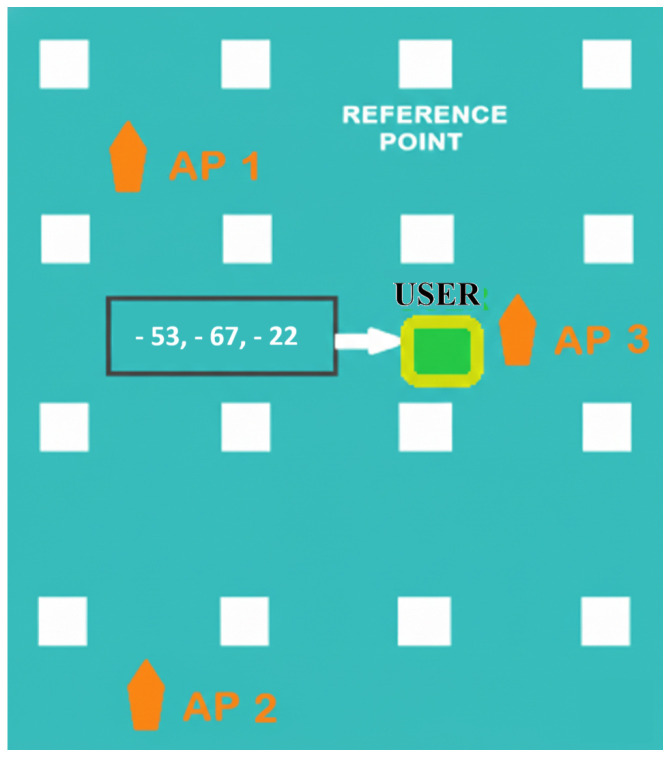
RSSI signature of user (target).

In this formulation, the likelihood $f(y|p_{j})$ accounts for randomness from noise and environmental changes (aleatoric uncertainty), while priors—when present—address uncertainty from limited data (epistemic uncertainty) [46]. MLE is effective when working with extensive fingerprint databases. As datasets grow larger and more complex, machine learning naturally becomes the preferred tool for prediction. In our work, we employ a multi-layer perceptron (MLP)—a feedforward neural network—for positioning. Figure 1 illustrates a user’s RSSI signature.

### 2.2. Reliability of LoRa-Based RSSI in Complex Environments

A significant block for RSSI-based indoor localization is the effect of multipath and shadowing [9], which makes path loss unpredictable, turning it into a random process and preventing any single mathematical model from precisely predicting RSSI at a specific location [47]. Consequently, traditional systems often need regular manual recalibration—a labor-intensive and time-consuming process to maintain up-to-date data.

LoRa relies on Frequency Shift Chirp Modulation (FSCM), which is based on the Chirp Spread Spectrum (CSS) method. When substantial bandwidth is available, CSS-based systems can actually distinguish and recombine multipath echoes, effectively minimizing flat fading [48]. Studies have demonstrated that CSS systems significantly reduce multipath interference in real-world conditions [49]. Thanks to this inherent resilience to interference and multipath fading [50], LoRa is particularly reliable for producing robust RSSI fingerprints. Additionally, the mathematical design of FSCM keeps hardware requirements straightforward, allowing it to handle multipath signals directly without needing a RAKE receiver [51]. LoRa’s resistance to these environmental factors remains strong across diverse radio environments as well [23].

In developing indoor localization for mobile robots, we deliberately selected the Received Signal Strength Indicator (RSSI) over Channel State Information (CSI). This was a practical decision: RSSI is simpler to implement and less prone to changes in the environment. While CSI-based Wi-Fi systems can reach decimeter-level precision, they face major challenges—severe phase instability and difficult deployment. Even a basic hardware restart can alter the phase enough to invalidate an entire fingerprint database, and CSI systems usually require many more reference points compared to RSSI-based approaches [52]. In our setup, the RSSI signature remains stable even where Wi-Fi or BLE readings fluctuate widely. By adjusting the spreading factor using SSIM and evaluating stability with Shannon entropy, we show that a LoRa-based RSSI system achieves high validation accuracy. This offers a robust, low-power alternative to the computational complexity and hardware issues found in CSI-based solutions [52].

**Figure 2:**
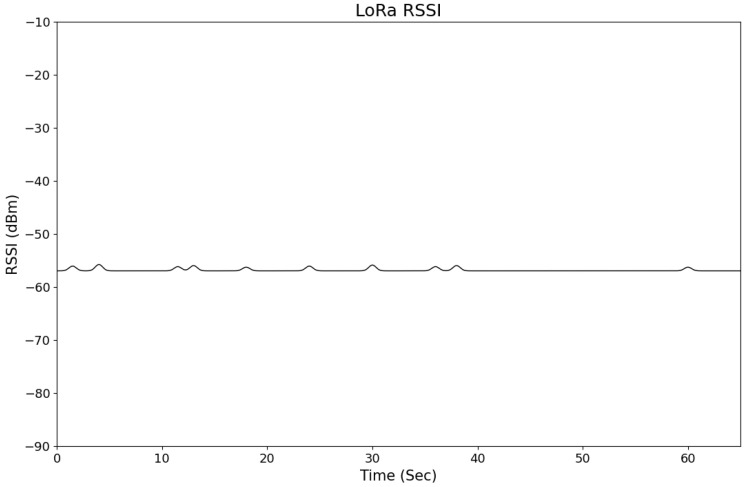
LoRa RSSI samples.

For our tests, we collected RSSI time-series measurements for 63 s, sampling every 100 milliseconds. The result is presented in Figure 2. Inside a congested lab environment, the RSSI from one access point varied very little (around 3 dBm). BLE can fluctuate by 30 dBm over 45 s, ZigBee by 9 dBm over 250 s, and WiFi by 7 dBm in just 30 s [53]. Our observations confirm that LoRa RSSI is much more stable. By using consistent time-series RSSI values for the purpose of fingerprinting, our position estimation remains reliable and robust [54].

Multi-modal systems such as HybridZone [55] have combined acoustic and Wi-Fi sensing for finer resolution. But, they introduce significant complexity, which demands hardware synchronization, increased sensitivity to background noise and Wi-Fi fading. These systems can achieve precise, local gesture recognition, but scaling them for large industrial spaces poses significant challenges. Our approach avoids these complications. We eliminate the need for high-frequency acoustic polling and specialized Wi-Fi NICs (Wireless Network Interface Controllers), making our system more resilient for tracking mobile robots in extensive, signal-dense environments. In addition to this, LoRa technology has several advantages over other wireless technologies, as discussed in the following Table 2.

## 3. RSSI Characterization and Preprocessing

Due to the stochastic nature of wireless transmission and small changes in the indoor environment, the received RSSI value from a particular Access Point (AP) sometimes produces a value that is out of the most observable range of RSSI data for that particular AP at some particular Reference Point (RP). Theoretical derivations and experimental research have shown that RSSI time-series data contains intrinsic fluctuations within bounded limits [26].

Although there is no standard path loss channel model for every type of indoor environment, the authors of [47] proposed a new channel model that takes into account several parameters like indoor wall thickness, angle of antenna orientation, height of receiver and transmitter antennas, values of attenuation factor at different angles of incident, etc., and they established their channel model by collecting empirical data. After predicting RSSI values with this model, the localization performance using the predicted RSSI fingerprint dataset attains prediction confidence of 33% at 3 m of distance and 78% at 9 m of distance [47], and it is certain that localization with the RSSI fingerprint predicted with this channel model is not deployable for a practical case. The reason for this is the noise associated with the RSSI data. This noise not only adds uncertainty to the RSSI data but also encrypts the relation of signal path loss in accordance to the complex indoor environment. Hence, RSSI preprocessing is a crucial stage at the time of recording the RSSI fingerprint, which is done by collecting a time series of RSSI samples over some period of time and then preprocessing it through stages in order to obtain a single RSSI value at a reference point. RSSI preprocessing techniques that are used in the reported work are given in the following subsections.

### 3.1. RSSI Preprocessing and RSSI Smoothing

As stochastic noise is present in indoor RSSI data, it makes accurate localization prediction significantly challenging. As shown in Figure 2, LoRa’s Chirp Spread Spectrum (CSS) modulation effectively handles flat fading. CSS modulation manages multipath echoes well in relatively stable environments. But, actual deployment sites are never static; the environment is constantly changing. These variations introduce non-Gaussian noise. This is where preprocessing becomes crucial. It enables the system to distinguish between the underlying signal and the random fluctuations caused by dynamic changes in the environment.

Machine learning models like Multi-layer Perceptron (MLP) are sensitive to minor variations in the training data, which can significantly impact their performance. To address this, we apply a Simple Moving Average (SMA) filter. This filter smooths out these small fluctuations, allowing the MLP to learn the stable spatial “signature” instead of memorizing incidental noise from a single measurement session.

Raw RSSI streams also capture unusual anomalies like hardware timing errors and packet collision, which manifest as outliers. Our pipeline incorporates an outlier detection step to ensure these anomalies do not contribute to the final fingerprint database. After outliers are removed, the RSSI data, though bounded, still contains substantial fluctuations. As the RSSI fluctuations still persist, before the averaging operation is performed to extract one single RSSI value from an AP to a particular RP, it is required to make the RSSI time series data smooth so that the effect of noise reflected by fluctuations in RSSI measurement can be reduced to some extent. In order to smooth the fluctuating RSSI data, the established technique is to apply the ‘moving average filtering’ operation to the data [64,65]. Simple Moving Average (SMA) filtering has been chosen for smoothing the received time series RSSI data at the time of the RSSI recording process. This RSSI preprocessing stage involves three tasks: outlier detection, RSSI smoothing and RSSI averaging. Training the MLP on this cleaned, averaged data, rather than the noisy raw samples, produces a noticeable reduction in Mean Square Error (MSE) during localization.

### 3.2. Entropy Analysis of RSSI Fingerprints

The presence of heavy shadowing due to obstructions and multipath propagation of signal within an indoor environment changes the received signal strength measurement at a particular reference point over different timestamps [66]. In this paper, the entropy of the fingerprint database has been calculated in order to quantify the randomness associated with the RSSI fingerprints. This was done in order to explore the useful arrangement of access points and receiver module so that relatively stable fingerprints can be collected. In this work, a set of fingerprints was collected by keeping access points and receiver antennas below the height of common furniture like tables, chairs and racks present in the indoor laboratory environment, then another set of fingerprints was collected by keeping the antennas above the furniture (2 m). The entropy was calculated for both sets of collected fingerprints. It was observed that the increase in height of antenna positions resulted in a decrease in the entropy value of the set of collected fingerprints. The entropy that was calculated for the first case was found to be 12.74. The entropy measured for the set of RSSI fingerprints, after increasing the antenna height, was found to be 5.79. This reduction in entropy value by 55% indicates that the elevated antenna height increased the LoS visibility; hence, relatively more stable fingerprints were collected because increased LoS visibility results in lesser variability in the RSSI values [67]. This approach of quantifying the randomness of RSSI fingerprint proved to be useful for exploring the proper arrangement of the experimental setup. Below, the steps of entropy calculation for a set of RSSI fingerprints are shown. From Equation (1), we know that ${r_{j}}^{i}\left(t_{m}\right)$ represents the RSSI at the *j*th reference point for the *i*th AP and at time instance $t_{m}$. Let us denote ${P_{j}}^{i}\left(T=t_{m}\right)$ as the probability mass function (PMF) of the RSSI samples recorded at time instances T = $t_{1}$, …, $t_{m}$ at *j*th RP for *i*th AP. The RSSI samples from each AP are collected at the receiver with a time division multiplexing approach, i.e., the collection of RSSI for any particular AP is totally independent of other APs. Therefore, the joint PMF of RSSI samples for different APs is the multiplication of each of the PMFs for all the APs. If we denote this joint PMF with $\bar{P}$, then

(10) $$\bar{P_{j}}=\prod_{i=1}^{L}P_{j}^{i}\left(T=t_{m}\right)$$

Therefore, the entropy of the RSSI signature at the *j*th reference point, denoted as $H_{j}$, is

(11) $$H_{j}=-\sum_{m=1}^{M}{\bar{p}}_{j}\left(t_{m}\right)\log_{2}{\bar{p}}_{j}\left(t_{m}\right)$$

We know that the entropy of a system consisting of several subsystems is the summation of the entropy of each subsystem. Therefore, the entropy of the whole set of RSSI fingerprints, denoted as H, is

(12) $$H=\sum_{j=1}^{N}H_{j}$$

From the above equations, the entropy of a set of RSSI fingerprints can be calculated.

## 4. System Architecture

**Figure 3:**
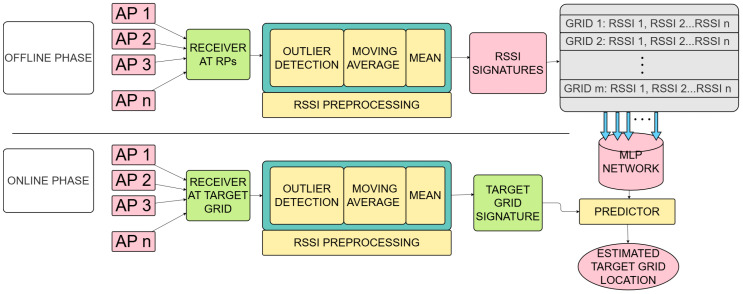
System architecture of LoRa RSSI fingerprint-based IPS.

The comprehensive system architecture is shown in Figure 3. As depicted in the framework, LoRa RSSI measurements from different access points (APs) are collected at a particular reference point (RP). Then, they are transmitted to a central processing unit (PC) via a dedicated LoRa connection. The measurements are organized on the PC as vectors in comma-separated value (*.csv*) files; each file contains multiple raw RSSI samples at each RP. After the implementation of the preprocessing stages, it yields a singular refined RSSI vector. This protocol is repeated across all marked indoor RPs. The workflow is managed through Python-scripted automation. After collecting enough of these vectors, one RSSI fingerprint is constructed. The procedure is repeated to assemble a robust and diverse database. Before using these fingerprints in the position estimation algorithm, the fingerprints are normalized.

Machine learning is applied for position prediction due to the nonlinearity mapping of RSSI fingerprints associated with spatial coordinates [68]. Matching RSSI fingerprints to precise locations is a highly complicated process, as indoor signals suffer from scattering and multipath propagation. Machine learning algorithms are found to be suitable at uncovering patterns in such data, especially in the case of non-Gaussian noise [69]. Moreover, ML architectures can manage voluminous datasets [70], and Bayesian probabilistic models can avoid misclassifications caused by outliers [46]. Altogether, ML is the best fit for handling the complex relationship between RSSI and spatial distance.

Rather than using deep learning (DL), which handles feature selection automatically [70], the reported work utilized a more straightforward route. The input of a shallow multi-layer perceptron (MLP) or feedforward artificial neural network is the preprocessed RSSI values from each access point.

The reported localization framework operates through a fully automated hardware–software pipeline. It ensures that RSSI preprocessing remains consistent in both offline and online training phases. During the online phase, the setup utilizes the SX1278 LoRa modem (Semtech Corporation: Camarillo, CA, USA) and ESP8266 (Espressif Systems: Shanghai, China) to collect 30 RSSI samples from each of the four LoRa anchors in one batch, as it provides statistically significant data for stepwise preprocessing in the Python script. First, we use quartile-based outlier detection to filter out any anomalous data or outliers. Next, a simple moving average (with a window size of 5 samples) is applied to smooth the data. Finally, the results are averaged to obtain a single four-element fingerprint vector. By keeping the preprocessing method identical in both the offline and online phases, it is ensured that the MLP always receives input features consistent with its training data. This consistency is crucial for maintaining reliable localization performance in practical settings.

### 4.1. Experimental Setup

**Figure 4:**
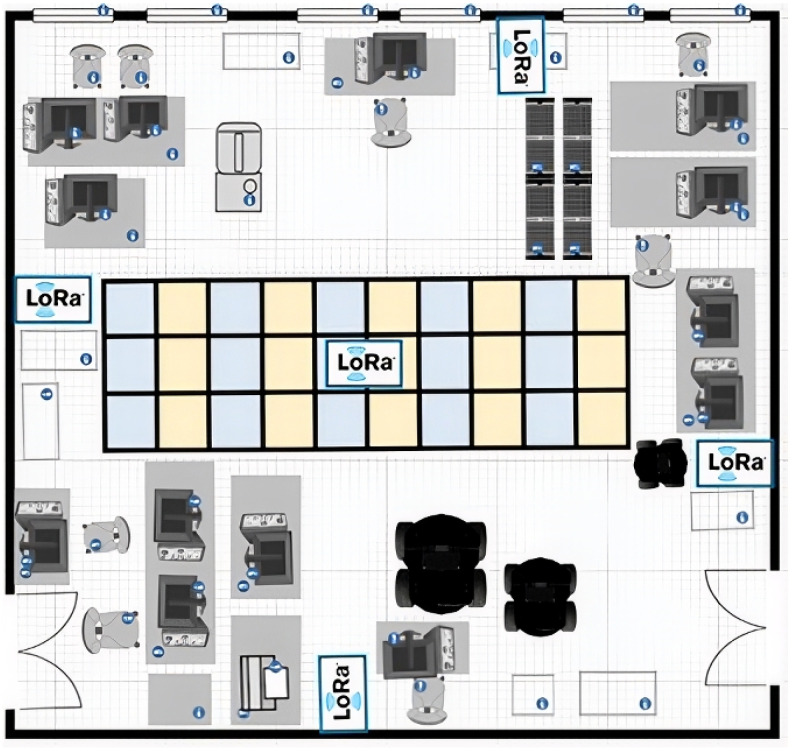
Congested indoor laboratory experimental setup.

The experiments were conducted in a dynamic and congested laboratory environment filled with metal furniture, desktop computers, and people moving about. It essentially produced multiple sources of electromagnetic interference mimicking the real-world environment. The strong interference and numerous obstacles usually disrupt signal measurements. For the localization tests, a rectangular area measuring 6 m by 1.8 m was designated, located in the center of a 10-by-10-meter room. To achieve high-resolution location mapping, the area was segregated into 30 equal squares, each measuring 0.6 by 0.6 m. This grid size was chosen to balance the details of the robot navigation and the practical limits of RSSI-based fingerprinting. Figure 4 illustrates the lab setup, highlights the localization area in the center, and indicates where the access points were placed.

The localization goal was to have the system identify the robot’s receiver and assign it to the correct cell on the spatial grid. The localization was treated as a multi-class classification task where the system learns to associate the received noisy RSSI readings with accurate location labels.

### 4.2. Configuration of LoRa Nodes

**Figure 5:**
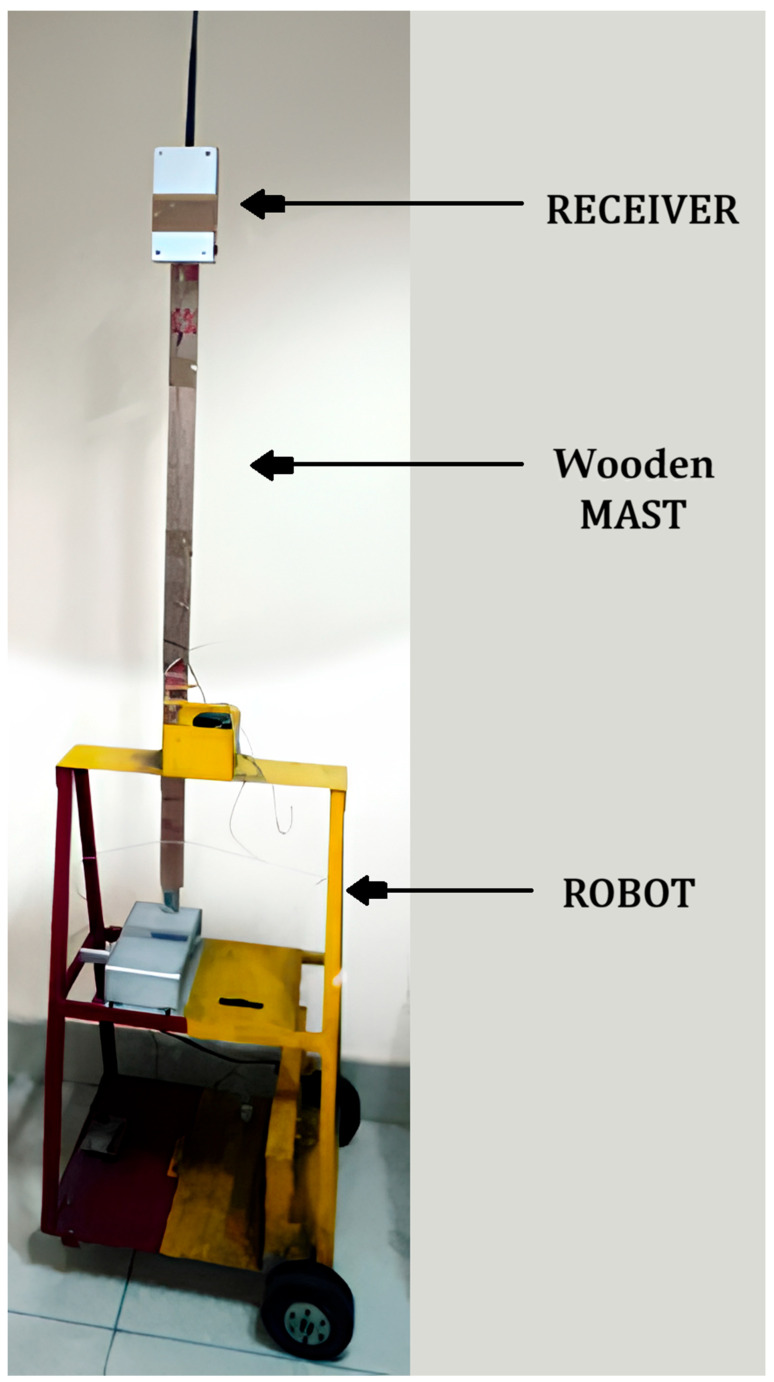
The mobile robot used in the experiment.

Four access points (APs) were installed throughout the experimental arena. Entropy analysis showed that signal fingerprints produce superior stability when antennas were positioned around 2 m above ground level. Therefore, all APs were mounted on the wall at this height. The receiver was mounted on a mobile robot, and the antenna was set at 2 m, held up by a wooden mast (Figure 5).

**Figure 6:**
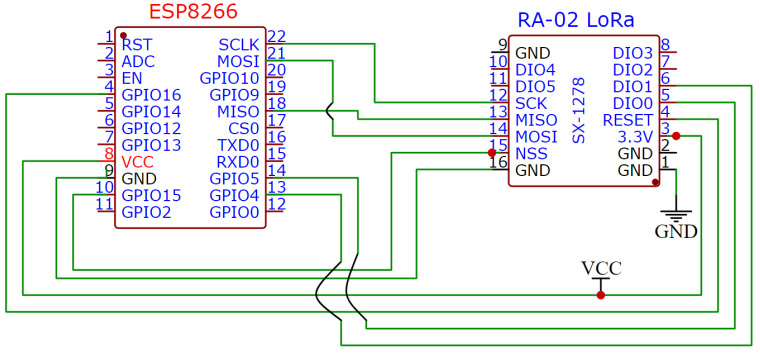
Schematic of ESP8266 with SX-1278.

LoRa communication was instantiated via SX1278 LoRa modems interfaced with ESP8266 NodeMCU modules, as schematized in Figure 6. The SX1278, a cost-effective RF transceiver, operates across 420–450 MHz with −136 dBm sensitivity, 1.8–3.7 V supply, and +20 dBm maximum transmit power. ESP8266 General-Purpose Input–Output (GPIO) pins interface SX1278 control lines; the latter draws 3.3 V from the onboard AMS1117 regulator. The ESP8266 features an 80 MHz clock, 64 KB RAM, and 4 MB flash, with CH340G USB-serial (Jiangsu Qinheng Microelectronics Co., Ltd., Nanjing, China) conversion for firmware upload. It is powered by an external 5 V DC source, and transmission is signaled via a GPIO-driven LED. LoRa communication was established using SX1278 modems interfaced with ESP8266 NodeMCU boards, as shown in Figure 6. The SX1278 is an affordable RF transceiver that operates in the range of 420 to 450 MHz with −136 dBm sensitivity, 1.8–3.7 V supply, and +20 dBm maximum transmit power. ESP8266 GPIO pins interface SX1278 control lines (Figure 6); the latter draws 3.3 V from the onboard AMS1117 regulator (Advanced Monolithic Systems, Livermore, CA, USA). The ESP8266 features an 80 MHz clock, 64 KB RAM, and 4 MB flash, with CH340G USB-serial conversion for firmware upload. It is powered by an external 5 V DC source, and transmission is signaled via a GPIO-driven LED.

For spreading factor (SF) optimization [51], two sets of RSSI fingerprints (at different times) were collected for SF values of 7, 10, and 12. This was done in 30 grid cells using three APs, resulting in a three-dimensional RSSI vector for each cell. The resultant RSSI strength maps were then rendered into images, and the Structural Similarity Index Measure (SSIM) [71] was applied to assess the similarity of fingerprint pairs over time.

**Figure 7:**
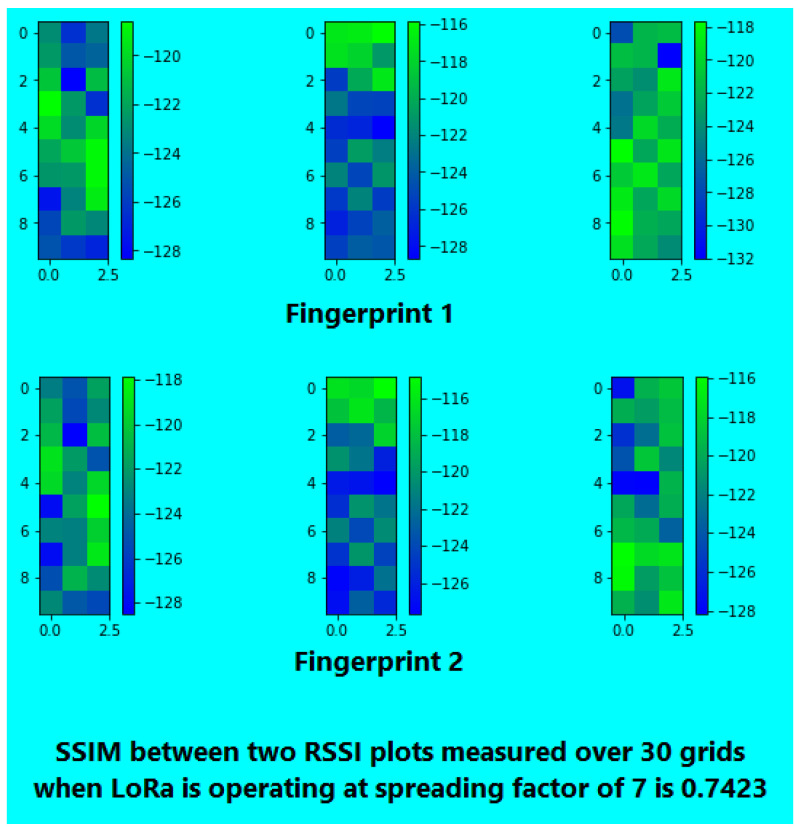
SSIM for SF 7.

**Figure 8:**
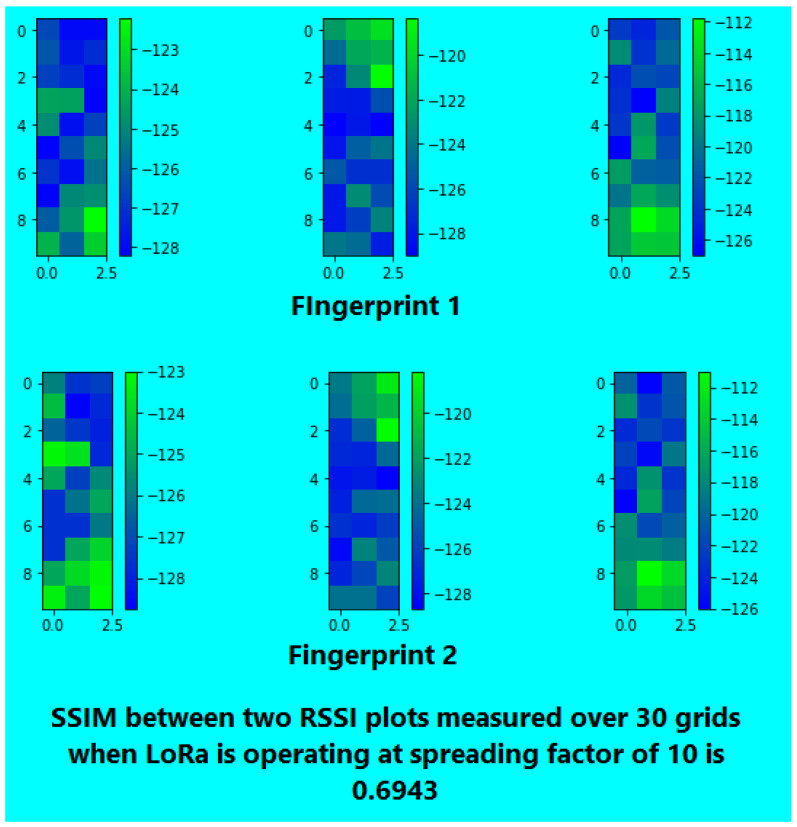
SSIM for SF 10.

**Figure 9:**
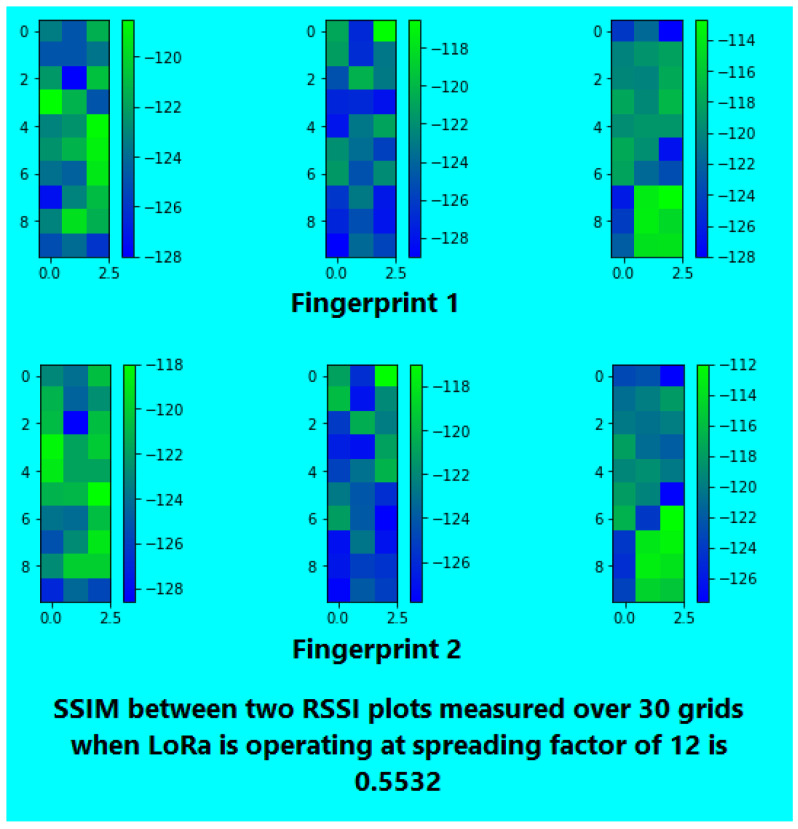
SSIM for SF 12.

After converting RSSI values to color-coded grids, SSIM was used to evaluate the structural similarity between pairs of temporal fingerprints at SF 7, 10, and 12 (see Figure 7, Figure 8 and Figure 9 ). SF 7 had the highest similarity; hence, it was used for the rest of our experiments.

**Figure 10:**
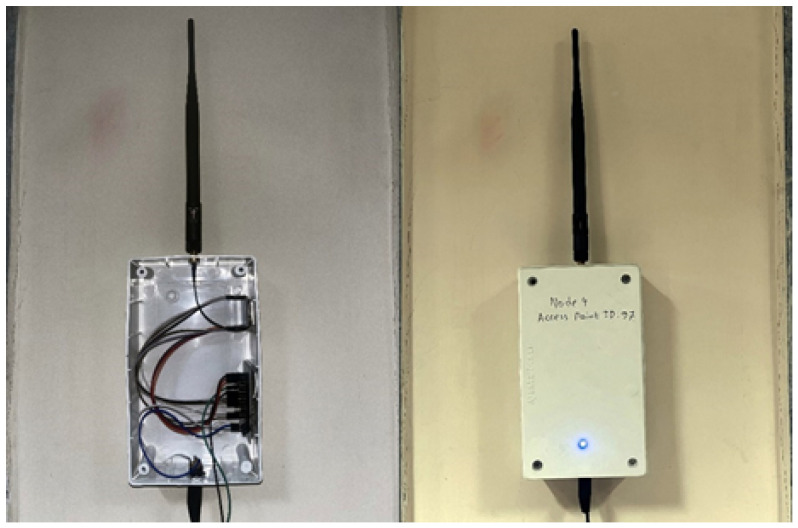
Access point nodes mounted on the wall.

**Figure 11:**
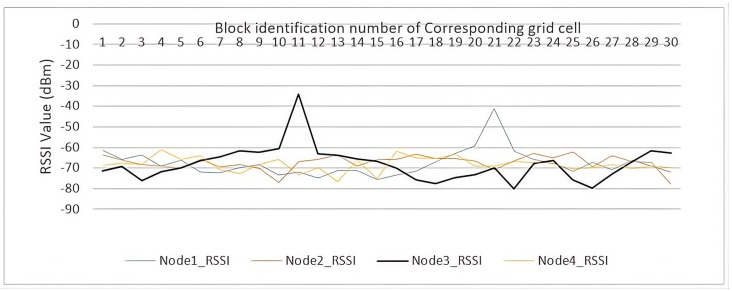
Graphical representation of a single RSSI fingerprint from the database.

For the hardware setup, all LoRa nodes operated with identical parameters: 128 kHz bandwidth, 4/8 coding rate, +2.0 dBm transmission power. A total of six LoRa nodes were used in the experiments: four wall-mounted APs (see Figure 10), one receiver mounted on the robot at a height of 2 m, and a sixth node connected to a PC for both offline fingerprinting and real-time prediction through USB serial communication. This extra node allowed for remote operation. The PC collected RSSI time series from each AP and reference point, cleaned the data following our RSSI preprocessing method, and combined the values into fingerprints as described in Equation (1). The receiver (the master) sent queries to the APs, which responded in sequence. We extracted RSSI values from their responses and sent all data to the PC for storage and analysis. For offline measurements, we collected RSSI fingerprints at the center of each grid cell with the receiver positioned centrally. Figure 11 provides an example of these fingerprints.

**Figure 12:**
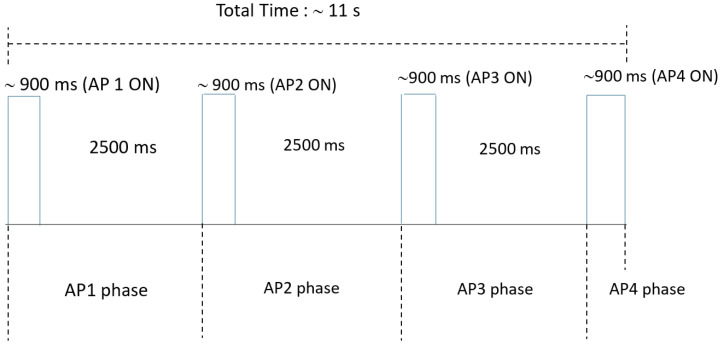
Representation of the transmission time cycle.

RSSI transmission payload was encoded into a single 8-bit packet. In this scheme, three bits were allocated for node identification, allowing unique addressing of up to 8 LoRa nodes ( In this work, 4 nodes are used), while the remaining five bits were dummy bits. The minimum transmission packet is preamble+sync+payload, and it takes around 30 ms to transmit. At a data rate of 3.5 kbps, the system collects 30 data signals from four APs. It requires around 900 ms to collect 30 packets from one AP. This is followed by a 2.5 s guard interval to settle down the RSSI resister and switch between one LoRa AP to ESP 8266 node MCU. This creates a total time cycle of approximately 11.1 s (as illustrated in Figure 12). The transmission time required by an AP to transmit 30 samples is around 900 ms. With the mentioned transmission time and 2.5 s guard interval, effectively around 10 RSSI samples are collected per second from one AP. The total data collection takes approximately 12 s. Adding one more AP means around 3.5 s more time to record the RSSI signature for a particular location. After around 90 s of waiting time, the next instance of sample collection is done, which makes the effective LoRa duty cycle 1%. The mobile robot operates at a nominal velocity of 5 cm/s. Over a period of 90 s and at such a low speed, accumulated incremental odometry errors are small. Moreover, for addressing the global localization and kidnapped robot problems, continuous high-frequency localization queries are not strictly necessary. Instead, periodic re-localization updates are sufficient to correct accumulated drift and re-position the robot’s pose within the global reference frame.

All the experiments were conducted in a busy 10 m × 10 m laboratory. While LoRa is typically used for long-range, low-power networks, we chose it for this local environment because of its physical layer advantages. Chirp Spread Spectrum (CSS) modulation is robust against multipath fading and interference, which are major concerns in cluttered indoor spaces. Additionally, LoRa’s sub-GHz signals penetrate obstacles like concrete and metal more effectively than 2.4 GHz alternatives.

### 4.3. MLP Network Architecture and Implementation

The positioning system is modeled as a multi-class classification problem (for 30 cells and 18 cells) instead of a continuous regression problem due to the following: The relationship between RSSI and distance is fundamentally stochastic. Multipath fading, shadowing, and random fluctuations [72,73] make the signal unpredictable and noisy. Using regression is not practically feasible under these circumstances [1].

On the other hand, classification offers a more effective solution. The multi-layer perceptron (MLP) outputs a probability distribution for every cell. Simply selecting the cell with the highest probability (maximum-likelihood), the global ambiguity is effectively resolved [74]. The result immediately produces a robust global observer. Through the classification method, the chaotic, nonlinear RSSI signals can be transformed into stable spatial grids, and the reliability of grid-based area coverage in autonomous navigation is enhanced [75].

Following this, the robot’s wheel odometry can then refine its position locally. At the core of this localization system is a feedforward MLP, specifically tuned for the stochastic, nonlinear RSSI patterns found indoors with LoRa. Being a shallow network, it is balanced for accurate classification and embedded deployment, as it is computationally lightweight enough to run on embedded hardware. The robot is able to execute the localization algorithm directly on its onboard processor; however, retraining and model training are done offline using a standard PC. The system is suitable to work on edge computing platforms, such as an Intel NUC or NVIDIA Jetson.

### 4.4. Architectural Topology and Hyperparameter Optimization

**Figure 13:**
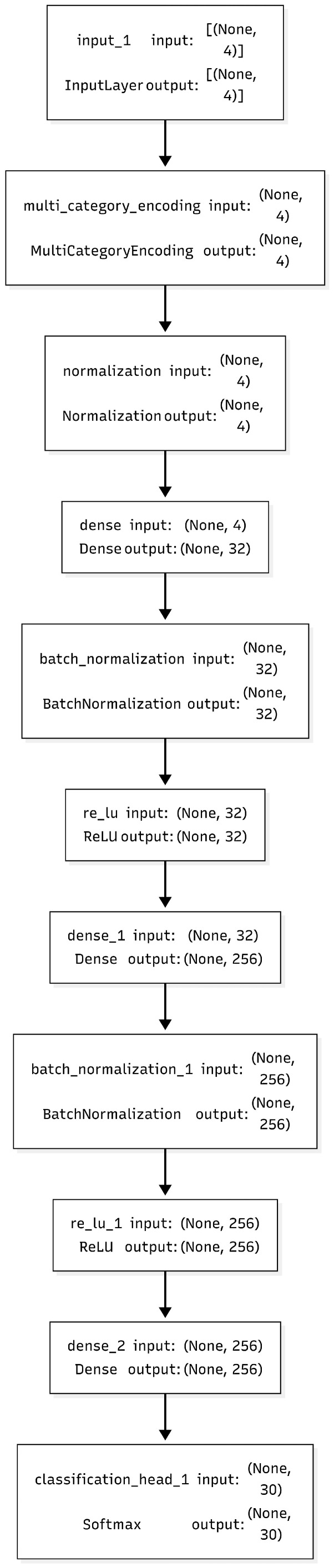
MLP network architecture.

The schematic diagram of the topology of the neural network architecture is shown in Figure 13. The first layer of the network is the input layer of L=4 neurons. Each neuron receives the RSSI reading from a separate access point. The next layer is a single hidden layer with 16 rectified linear unit (ReLU) neurons. The ReLU neuron layer performs nonlinear feature extraction and abstraction. The final layer is an output layer with 30 softmax neurons. This layer is responsible for encoding the probability of the grid cell. ReLU, defined as f(z)=max(0,z), performs sparse activations and helps prevent vanishing gradients. The softmax function ensures the output probabilities across the 30 grid cells sum to one, $\sum_{c=1}^{30}{\hat{y}}_{c}=1$, and it maintains the interpretability of the model’s predictions. For the hyperparameter optimization, an exhaustive grid search was conducted (over $G\in R^{\left|G\right|=3^{5}\times 3^{3}=2187}$ configurations). The optimization was performed with the following details: It was conducted over hidden units {8, 16, 32}, optimizers (Adam, RMSprop, SGD), activations (ReLU, tanh, LeakyReLU), batch sizes {32, 64, 128}, dropout rates {0.0, 0.2, 0.5}, and L2 penalties $\left\{10^{-5},\;10^{-4},\;10^{-3}\right\}$. Furthermore, each combination was evaluated using 10-fold stratified cross-validation on 90% of the training set. The categorical cross-entropy was also minimized with L2 regularization:

$$L\left(\theta \right)=-\sum_{i=1}^{N}\sum_{c=1}^{30}y_{i,c}\log\left({\hat{y}}_{i,c}\right)+\lambda \sum_{l}{∥W^{\left(l\right)}∥}_{2}^{2},$$

where $y_{i,c}$ is the one-hot encoded label for sample *i* and class *c*, and ${\hat{y}}_{i,c}$ is the predicted probability. Here, θ represents all trainable weights and biases, and $\lambda =10^{-4}$ represents the regularization parameter or the regularization coefficient. The optimal configuration has the following details: 16 ReLU units, the Adam optimizer (with $\beta_{1}=0.9$, $\beta_{2}=0.999$, $\epsilon =10^{-8}$, and initial learning rate $10^{-3}$), a batch size of 64, dropout rate p=0.2, and up to 800 training epochs with early stopping (patience = 50, $\Delta =10^{-4}$). ReduceLROnPlateau was also employed with factor = 0.5 and patience = 20 in order to halve the learning rate if validation performance plateaued for 20 epochs. This setup achieved satisfactory validation accuracies. For the initial 18-block study, an accuracy of 91.39% was achieved. For the total arena with 30 blocks, the setup achieved 91.80% accuracy, where test losses were stabilized at approximately L≈0.12. The neural network model was implemented using Keras’s Sequential Application Programming Interface (API) atop TensorFlow 2.15, running in the environment of Python 3.10. The network consists of a dense layer with 16 ReLU units, L2 regularization ($\lambda =10^{-4}$), expects four input features, applies dropout (p=0.2), and concludes with a 30-unit softmax output. The network was compiled with the Adam optimizer (learning rate 0.001), categorical cross-entropy loss, and monitored accuracy during training. This design completes direct and supervised classification of normalized RSSI feature vectors from input to output.

## 5. Results and Discussion

In the reported work, we have developed a LoRa RSSI fingerprint-based indoor positioning system. It employs a supervised multi-layer perceptron (MLP) classifier to associate chaotic RSSI signals with specific spatial locations. In this approach, the problem was considered as a multi-class classification task. The goal was to determine the location of a mobile robot inside of 30 grid cells (each measuring 0.6 by 0.6 m) within a 6 by 1.8 m rectangular area. Collecting RSSI fingerprints for every cell is labor-intensive. Hence, experimental validation was performed in two stages. Initially, the system was tested on a smaller 18-cell section (3 m × 1.8 m). After successful and satisfactory results were obtained in the smaller grid area, the coverage was expanded, and the entire area of 30 grid cells was included in the experiment.

**Figure 14:**
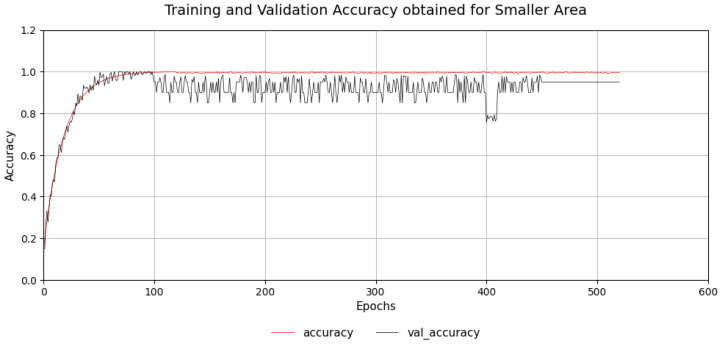
Training and validation accuracy of MLP classifier for 18-cell subdomain.

**Figure 15:**
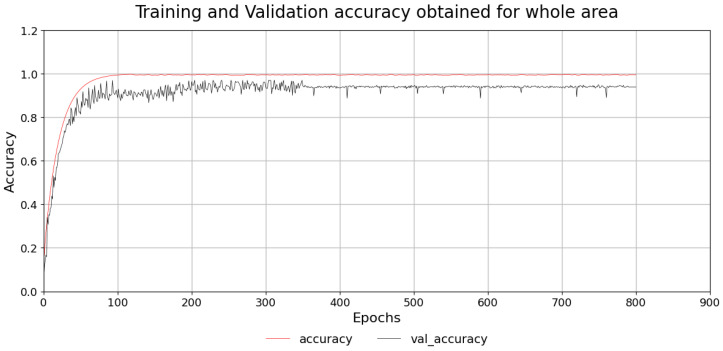
Training and validation accuracy of MLP classifier for 30-cell deployment.

RSSI fingerprints was collected for the first 18 cells, and the neural network training was done. The MLP achieved steady training and validation accuracy, which is illustrated in Figure 14. Subsequently, in the next set of experiments, using data from all 30 cells, the network was trained, which displayed stable convergence (refer to Figure 15). This confirms that the model’s architecture was optimal, appropriate and avoided overfitting.

**Figure 16:**
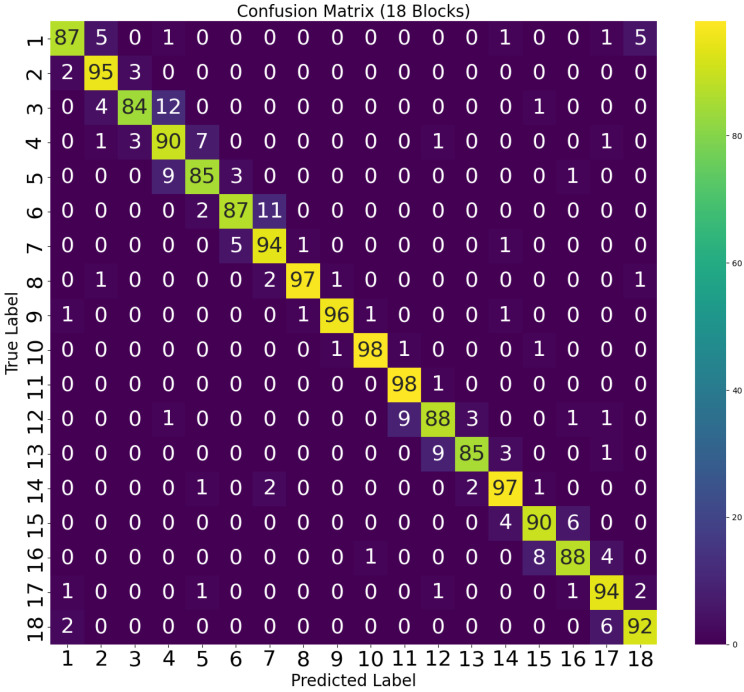
Confusion matrix for MLP multi-class classifier (18 cells).

**Figure 17:**
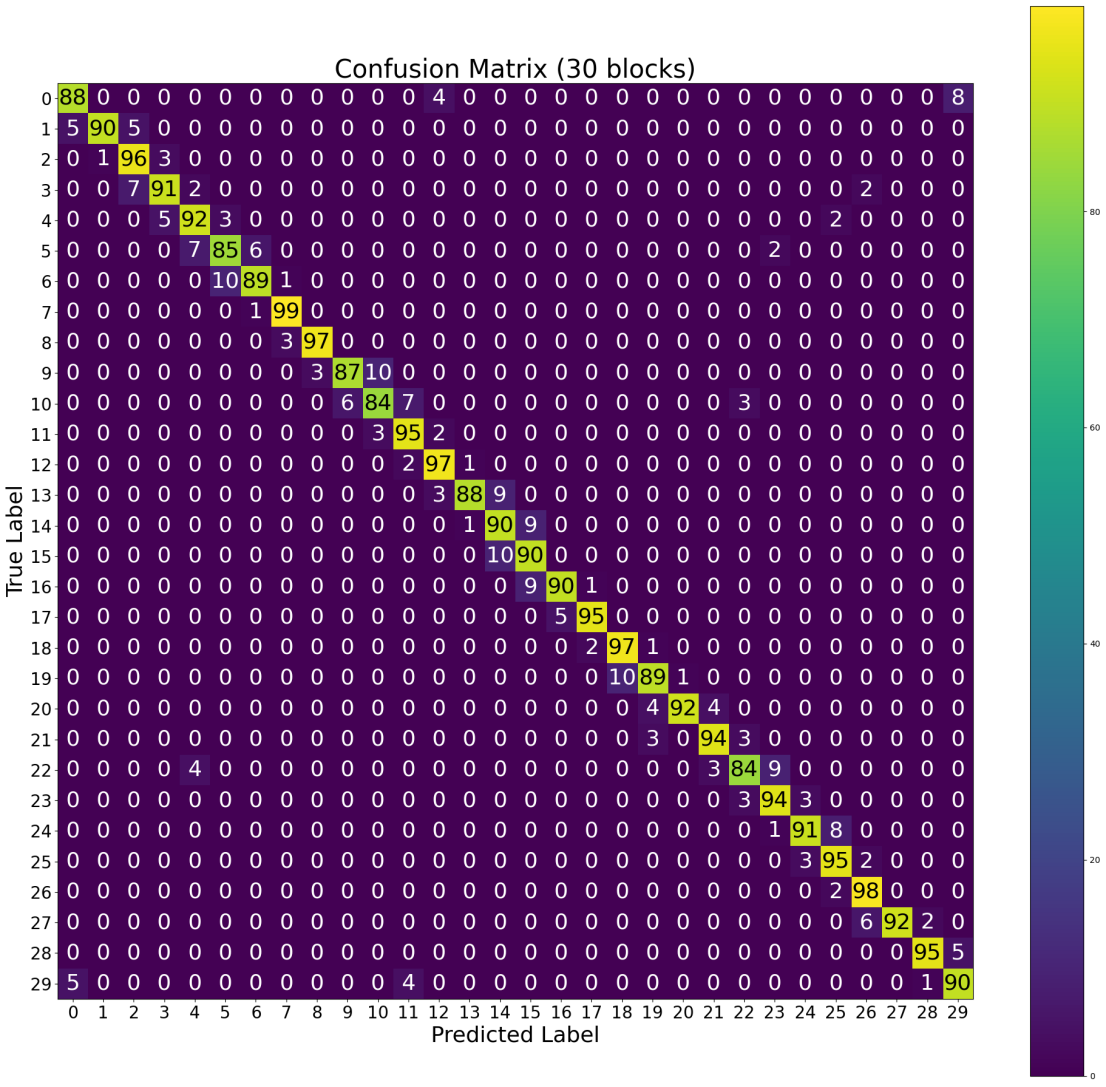
Confusion matrix for MLP multi-class classifier (30 cells).

The obtained confusion matrices for the 18-cell and 30-cell configuration experiments are shown in Figure 16 and Figure 17, respectively. In these confusion matrices, the actual class is represented by the rows, and the columns indicate the predicted class. An important metric, the true positives, is associated with the diagonal values. It indicates the instances where the model made correct predictions. On the other hand, off-diagonal values, just above or below, represent false positives and false negatives. Strong diagonal values with minimal off-diagonal entry are the characteristic trait of a good classifier. In this case, both matrices show prominent diagonal lines, and most of the errors appear in adjacent cells. This pattern matches the way RSSI gradients vary spatially. The confusion matrices demonstrate that the classifier is able to distinguish locations with high spatial accuracy.

Table 3 presents a concise comparison with other prominent LoRa RSSI localization methods that employ various other algorithms. This provides an assessment of how the lightweight MLP-LoRa IPS discussed in this paper compares to other approaches in the field.

**Figure 18:**
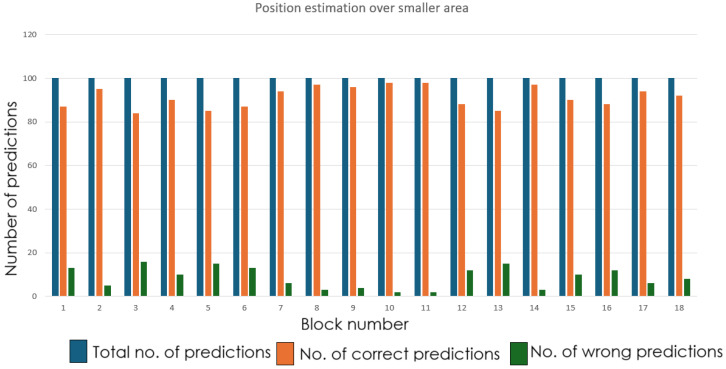
Prediction performance of LoRa RSSI-based IPS: 18-cell subdomain.

**Figure 19:**
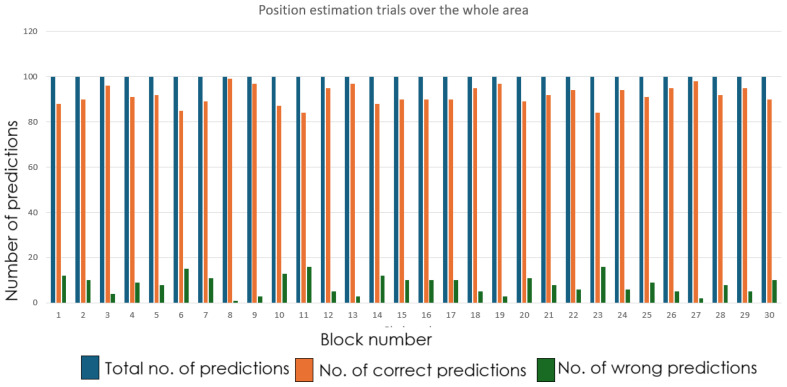
Prediction performance of LoRa RSSI-based IPS: 30-cell deployment.

**Figure 20:**
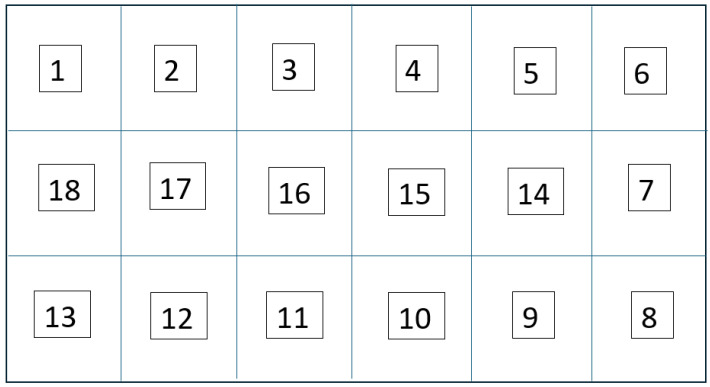
Labeling of the 18 grid cells.

**Figure 21:**
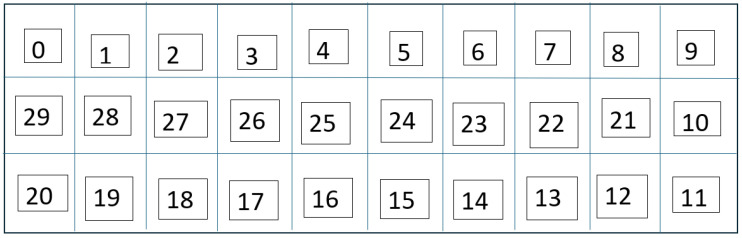
Labeling of the 30 grid cells.

The real-world localization performance was validated by positioning the robot with the receiver node at random grid centers. Figure 18 and Figure 19 demonstrate the accuracy of the predictions of the grid cells with the proposed LoRa RSSI-based IPS in estimating positions within the 18-cell subdomain and the 30-cell domain, respectively. In both tests, the reported system reliably identified grid-level locations with a resolution of 60 cm. The system performed well, even within the congested laboratory setting. The grid number labeling is shown in Figure 20 and Figure 21 for the 18-cell and 30-cell setups, respectively.

**Figure 22:**
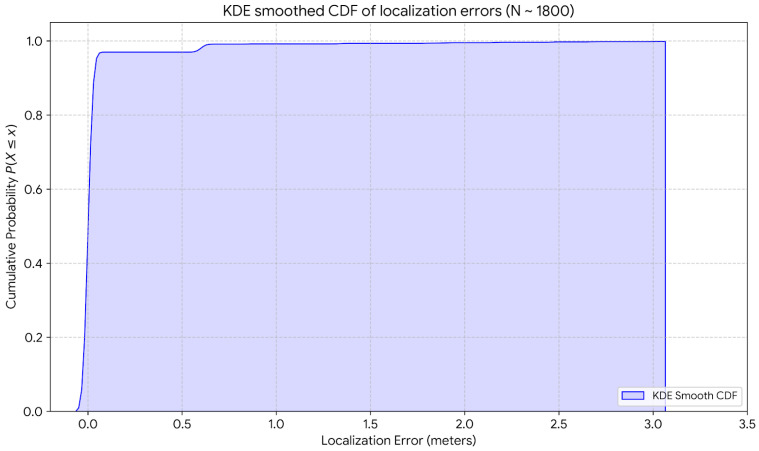
Kernel density estimation-smoothed Cumulative Distribution Function (CDF) of localization error for 18-cell configuration.

The Kernel Density Estimation (KDE)-smoothed Cumulative Distribution Function (CDF) of localization error for the 18-cell configuration (Figure 22) shows the precision of the reported localization system across around 1800 samples in the 18-grid arena. In the context of localization, more than 95% of the error is less than or equal to 0.6 m. Beyond this point, the distribution shows a few occasional outlier events, where localization errors reach a maximum of around 3 m.

**Figure 23:**
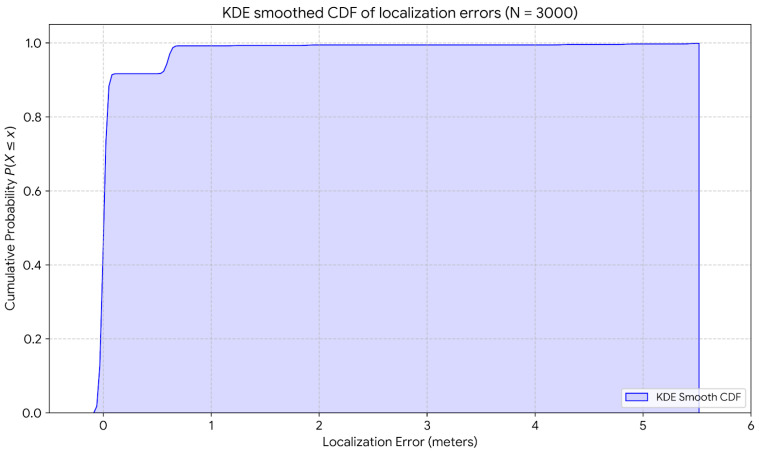
Kernel density estimation-smoothed Cumulative Distribution Function (CDF) of localization error for 30-cell configuration.

In the case of the 30-grid area, the KDE-smoothed Cumulative Distribution Function (CDF) plot in Figure 23 illustrates the precision of the reported localization system across approximately 3000 different samples. From the localization point of view, more than 96% of the error is less than or equal to 0.6 m. Beyond this point, the curve shows very few outlier errors, which reach up to 5.43 m.

The primary objective of the reported work is the localization of a radiation monitoring mobile robot. It is not motivated by precision manipulation tasks or docking. For radiation monitoring, a localization accuracy of 60 cm is technically reasonable and sufficient for reliable radiation detection operation. In the case of radiation monitoring, especially for gamma radiation, the spatial propagation characteristics are justified by grid cell-level localization. As gamma rays in air can travel far beyond 60 cm without attenuation, radiation intensity monitoring is not highly sensitive to sub-centimeter variations in position. For this purpose, a localization resolution of 60 cm × 60 cm grid cells is sufficient to identify the radiation contaminated zones and to perform spatial radiation mapping. The proposed system can perform high-level autonomous navigation. The reported system is deliberately designed to provide coarse but reliable cell-level localization, as continuous coordinate estimation is not a necessary requirement for the aforementioned purpose.

It is found that RSSI-based fingerprinting can not offer localization with sub-centimeter precision but it is highly reliable for region identification. For finer resolution, once the grid cell is identified using the proposed method, odometry-based dead reckoning of the robot IMU is fused and utilized to estimate the robot’s precise position within the identified cell.

In the configuration reported in this work (SF7, bandwidth 125 kHz, four access points), a complete online localization cycle starting from the reception of four RSSI measurements to preprocessing along with MLP inference requires approximately 100–150 ms. The computational processes of this pipeline, i.e., outlier removal, simple moving-average filtering, normalization and a forward pass through the 4–16–30 MLP, is performed in less than a few ms on a central processing unit (CPU). The experimental results show that the average localization estimation time, once the online sampling data collection is done, lies on the order of milliseconds.

In the reported RSSI fingerprinting-based localization method, the overall adaptation time is predominated by the offline stage. In the offline stage, a new fingerprint database needs to be collected, and Artificial Neural Network (ANN) retraining is required. Periodic recalibration (e.g., once or twice per week) is required to account for the intrinsic stochastic nature of the wireless channel and changes in the experimental environment. Periodic recalibration requires re-recording of the RSSI fingerprints at all the reference grid cells and retraining of the shallow MLP on the updated database. As the network size is small (4–16–30) and the dataset size is moderate, full retraining from scratch can be done in under a few minutes. The training was done using an Intel Core i7-11700T (11th Gen “Rocket Lake-S”) desktop processor with a base clock of 1.4 GHz. The CPU performance is comparable to the edge hardware like NVIDIA Jetson series (the Orin Nano or Orin NX) and INTEL NUCs (the NUC 11 or 12 Pro). Hence, adaptations to new environments or new obstacle layouts mainly incur short and offline retraining periods.

These outcomes establish LoRa RSSI fingerprinting as viable for sub-meter indoor localization, rivaling conventional Wi-Fi paradigms while leveraging narrowband stability advantages.

### Limitations of the System

The reported system does not output continuous coordinates, but it provides coarse location information, which is adequate for high-level navigation and area-based task planning. It is acknowledged that RSSI-based fingerprinting alone may lack the sub-centimeter precision required for complex maneuvering tasks. Consequently, this reported architecture provides a hierarchical localization pipeline:

- Grid-Level Localization (RSSI): As the RSSI fingerprinting is effective for region identification, it acts as a robust “global observer,” which narrows down the robot’s position to a specific grid cell (60 cm × 60 cm). This reduces the probability of multimodal uncertainty (multiple potential locations) in the domain of the robot kidnapping problem.
- Intra-Grid Precision (Odometry): Once the grid position is established, the odometry output from the robot’s IMU is fused to pinpoint the exact coordinates within that cell whenever that information is required.

This dual-stage localization approach, using RSSI for global grid-cell identification and odometry for local dead-reckoning, creates a robust solution to the localization problem. Here, we have reported the LoRa RSSI fingerprint-based localization only for the high-level robot navigation scenarios.

Furthermore, the reported system depends on a multi-stage preprocessing pipeline, i.e., simple moving average smoothing and statistical averaging, requiring the collection of a minimum number of samples to create an RSSI fingerprint with a high confidence level. As the velocity of the robot increases, the number of samples per “grid cell” decreases. In the field of robot localization, to address the Robot Kidnapping Problem and initial global localization in the map, the robot is programmed to stop briefly or to move with a significantly low velocity. This allows the reported system to accumulate a sufficient number of RSSI samples. It ensures that the fingerprint matched against the database is derived from a statistically significant sample size, which minimizes the probability of a location mismatch. We have identified a practical reliability threshold based on our hardware’s packet reception rate. As the robot moves from one center to the next center of the grid, beyond a velocity of approximately 5 cm/s (for area coverage and radiation mapping application, robots are required to move very slowly to collect radiation data samples using radiation detectors, which is the target use case of our localization method), a sufficient number of samples cannot be collected, which leads to degradation in MLP prediction accuracy. Because of this, the system is suitable for initial global localization, but the kidnapped robot problem persists, and correction of odometry localization error is necessary after a certain interval of time when sufficiently high amount odometry error is accumulated. Furthermore, in the reported work, LoRa is used for occasional global position corrections, not for very fast, continuous tracking, keeping us within the LoRa duty cycle limits. For one robot, a localization update works like this: the robot sends a short request, and four LoRa access points (APs) each send back short replies. At SF7 with 1 byte payloads, the total airtime used by each node in one update is 900 ms, so if the robot asks for a new position every 90 s, the transmit time per node stays well below the usual 1% duty cycle limit in sub-GHz ISM bands. High-rate motion inside a 60 cm cell is handled by odometry, so LoRa is only needed occasionally to correct odometry drift or to solve the kidnapped robot problem.

## 6. Conclusions and Future Scope

The efficacy of a LoRa-based Indoor Positioning System (IPS) for high-level robotic self-localization is presented in this work. By using the intrinsic stability of Chirp Spread Spectrum (CSS) modulation, the reported framework achieved sub-meter localization resolution (60 cm). The system utilized a computationally efficient, lightweight, shallow Multi-Layer Perceptron (MLP) architecture suitable for edge AI hardware deployment. An important methodological contribution of this work is the inclusion of Shannon entropy to quantitatively assess the randomness of RSSI fingerprints. This metric showed empirical verification that antenna position significantly reduces signal variability and enhances the accuracy of the spatial database.

Another important point of discussion is that while standard LoRa deployments generally rely on a centralized gateway, here, the peer-to-peer configuration was chosen for autonomous and infrastructure-independent localization. The mobile node acts as master, and it initiates the ranging process and collects RSSI data directly from fixed reference anchors. Using this decentralized approach, communication latency was significantly reduced, as it eliminated the requirement for a network server backhaul, which resulted in faster coordinate calculation.

The system successfully achieved a 91.8% classification accuracy in a congested laboratory environment, with 96 % localization errors within 0 to 0.6 m. Still, several scopes for further enhancement of the system have been identified. Future contributions of this work will involve autonomous mobile robots with simultaneous localization and mapping (SLAM) capabilities to automate the fingerprinting process, which will enable rapid system calibration and deployment.

This study showed high precision in a localized environment, but scaling the system to a much wider-area LoRa deployment would cause a trade-off between area coverage and localization resolution. In the case of long-range scenarios, RSSI leads to increased spatial uncertainty due to the logarithmic nature of RSSI. Furthermore, the requirement of higher Spreading Factors (SFs) for long-range communication would increase time-on-air, which might limit the update frequency of the localization algorithm. Hence, future iterations of this work could incorporate Time Difference of Arrival (TDoA) along with RSSI in order to maintain accuracy. Over large-scale distances, the proposed approach can leverage the system’s current fusion logic to mitigate large-scale shadowing effects.

Furthermore, we intend to study the effects of advanced environmental modifications such as Intelligent Reflective Surfaces (IRSs) and specialized radio-absorbing materials on signal stability. The environmental modifications are expected to mitigate the multipath interference and minimize the entropy of the RSSI signatures. Systematic testing of various spatial arrangements of these materials and the optimal configurations for ultra-stable indoor RF propagation can be deduced.

On the algorithmic side, future research requires the transition from static classifiers to sequential models, like Recurrent Neural Networks (RNNs) or Long Short-Term Memory (LSTM) networks. By using the temporal dependencies and previous state information of the mobile robot, these models are expected to significantly reduce the misclassification rates. This approach can also provide smoother and more coherent trajectory identification. Ultimately, the fusion of LoRa RSSI signatures with predictive temporal neural network models will provide a reliable, robust and low-power solution for the next generation of Industry 4.0 autonomous systems.

## Figures and Tables

**Table 1:** Works related to RSSI-based IPS.

Author	Description	Remarks
Lim et al. [19]	Utilized online RSSI measurements between APs using 802.11 standard to build IPS.	Experimented in zero configuration & explored the RSSI variance due to temporal & spatial variations of indoor environment.
Islam et al. [20]	Compared the RSSI measurements taken from LoRa, WiFi & BLE technology.	LoRa-RSSI was found to be more stable than WiFi & BLE-based RSSI.
Wixted et al. [21]	Wireless coverage of LoRa and LoRaWAN was explored.	The LoRa network coverage was reported to be wide enough even for problematic areas.
Anjum et al. [22]	Explored feasibility of LoRa technology for RSSI based IPS.	Machine learning-based position prediction algorithms for LoRa were reported to be excellent for IPS.
Staniec et al. [23]	Performance of LoRa was tested under heavy interference & heavy multipath conditions.	LoRa, under certain operational ranges of radio parameters, provides immunity to heavy multipath propagation & variable interference.
Wang et al. [24]	A novel deep learning-based position prediction algorithm termed DeepFi was proposed.	DeepFi was claimed to be capable enough to reduce localization error.
Ali et al. [25]	LoRa RSSI-based IPS utilizing a deep learning algorithm.	The IPS performance was reported satisfactory within an indoor environment.
Luo et al. [26]	Proposed adaptive wireless IPS through dynamic fingerprint collection by self-locating mobile robot.	Autonomous fingerprint collection helped maintain the IPS effectively.

**Table 2:** Comparative performance analysis of wireless technologies for indoor positioning systems.

Metric	ZigBee	WiFi	LoRa	BLE	LoRa Advantage
Power	30 mA Tx 1 µA standby [56]	400 mA Tx 20 mA standby [56]	28–44 mA Tx 1.4 mA standby [56]	40 mA TX 0.2 mA standby [56]	Lowest TX current
Path Loss	75.8–80.5% [57]	79–86% [20]	85–96% [20]	55–79% [20]	Best log-distance fit
Coverage	10–50 m [58,59]	10–100 m [58,59]	5–10 km LOS 0.5–2 km NLOS [58,60,61]	100 m [58,59]	Maximum NLOS range
Sensitivity	−100 dBm [62]	−95 dBm [62]	−137 dBm [63]	−95 dBm [62]	Superior sensitivity

**Table 3:** LoRa RSSI localization studies with multiple ML algorithms and position of the proposed lightweight MLP-LoRa IPS.

Study/Method	Tech. & Setup	ML Algorithms	Key Findings	Comments
Anjum et al. [22]	LoRa RSSI ranging and localization (indoor/outdoor)	SVM, regression splines, decision trees, ensembles	Ensembles outperform basic regressors but remain error-prone under multipath scenarios and are heavier for IoT devices.	Motivates using models with a good accuracy–complexity trade-off instead of computationally expensive ensembles.
LoRa fingerprinting with PSO–RF–FPL [76]	Indoor LoRa fingerprinting with multi-gateway setup	Random Forest + PSO, Gaussian/median filtering, Kriging	PSO-tuned RF improves accuracy but requires meta-heuristics and hybrid features, increasing system complexity.	Shows that higher accuracy is possible with complex pipelines that are less suitable for constrained edge deployments.
ML-based LoRa localization (multi-gateway) [77]	LoRa localization in indoor/industrial scenarios using several gateways	*k*NN, Random Forest, related classifiers	RF generally outperforms K-NN, yet both are sensitive to multipath scenarios and need careful parameter tuning.	Underlines the importance of robust preprocessing; our entropy and SSIM steps explicitly stabilize fingerprints before learning.
This work: entropy-stabilized MLP-LoRa IPS	LoRa RSSI fingerprinting in a congested lab, single robot in 6 m × 1.8 m (30 cells of 0.6m)	Shallow MLP (4 inputs, 16 ReLU units, 30-way softmax)	91.8% grid-cell accuracy using four RSSI features with entropy-based antenna placement and SSIM-based SF selection.	Deliberately chosen as a lightweight edge-AI model: sub-millisecond inference on embedded platforms while retaining sub-meter resolution.

## Data Availability

The data supporting the findings of this study will be made available in a GitHub repository following an embargo period of 6 months to allow for the commercialization of research findings.

## Abbreviations

The following abbreviations are used in this manuscript:

RSSI	Received Signal Strength Indicator
LoRa	Long-Range
SSIM	Structural Similarity Index Measure
MLP	Multi-layer Perceptron
IPS	Indoor Positioning Systems
LBS	Location-Based Services
GNSS	Global Navigation Satellite Systems
GPS	Global Positioning System
AGPS	Assisted Global Positioning System
PMF	Probability Mass Function
ReLU	Rectified Linear Unit
IMUs	Inertial Measurement Units
LoS	Line-of-Sight
VLC	Visible Light Communication
RF	Radio Frequency
LDA	Linear Discriminant Analysis
ToA	Time of Arrival
MSE	Mean Square Error
AoA	Angle of Arrival
PoA	Phase of Arrival
BLE	Bluetooth Low Energy
RSS	Received Signal Strength
CSI	Channel State Information
APs	Access Points
ML	Machine Learning
RP	Reference Points
MLE	Maximum Likelihood Estimation
ANN	Artificial Neural Network
FSCM	Frequency Shift Chirp Modulation
CSS	Chirp Spread Spectrum
SMA	Simple Moving Average
DL	Deep Learning
GPIO	General-Purpose Input–Output
API	Application Programming Interface
MAP	Maximum a Posteriori
LED	Light-Emitting Diode
K-NN	k-Nearest Neighbor
SVM	Support Vector Machine
FP	False Positive
FN	False Negative
TP	True Positive
CDF	Cumulative Distribution Function
LoRaWAN	Long-Range Wide-Area Network
SLAM	Simultaneous Localization and Mapping
IRS	Intelligent Reflective Surfaces
RNNs	Recurrent Neural Networks
LSTM	Long Short-Term Memory
CPU	Central Processing Unit
KDE	Kernel Density Estimation

## References

[1] Zafari B., Gkelias A., Leung K.K. (2019). A survey of indoor localization systems and technologies. IEEE Commun. Surv. Tutor..

[2] Wang X., Mao S., Pandey S., Agrawal P. (2014). CA2T: Cooperative antenna arrays technique for pinpoint indoor localization. Procedia Comput. Sci..

[3] Liu H., Darabi H., Banerjee P., Liu J. (2007). Survey of wireless indoor positioning techniques and systems. IEEE Trans. Syst. Man Cybern. Part C (Appl. Rev.).

[4] Umetani T., Kondo Y., Tokuda T. (2020). Rapid development of a mobile robot for the nakanoshima challenge using a robot for intelligent environments. J. Robot. Mechatron..

[5] Lee T.-J., Kim C.-H., Cho D.-I.D. (2018). A monocular vision sensor-based efficient SLAM method for indoor service robots. IEEE Trans. Ind. Electron..

[6] Wang K., Ma S., Chen J., Ren F., Lu J. (2020). Approaches, challenges, and applications for deep visual odometry: Toward complicated and emerging areas. IEEE Trans. Cogn. Dev. Syst..

[7] Esfahani M.A., Wang H., Wu K., Yuan S. (2019). AbolDeepIO: A novel deep inertial odometry network for autonomous vehicles. IEEE Trans. Intell. Transp. Syst..

[8] Wang H., Li J., Cui W., Lu X., Zhang Z., Sheng C., Liu Q. (2019). Mobile robot indoor positioning system based on K-ELM. J. Sens..

[9] Kim Geok T., Zar Aung K., Sandar Aung M., Thu Soe M., Abdaziz A., Pao Liew C., Hossain F., Tso C.P., Yong W.H. (2020). Review of indoor positioning: Radio wave technology. Appl. Sci..

[10] Huang J., Junginger S., Liu H., Thurow K. (2023). Indoor Positioning Systems of Mobile Robots: A Review. Robotics.

[11] Ergul O., Dinc E., Akan O.B. (2015). Communicate to illuminate: State-of-the-art and research challenges for visible light communications. Phys. Commun..

[12] MaxBotix Inc MaxBotix Ultrasonic Sensors. https://maxbotix.com/?srsltid=AfmBOoq8WYwqGOE0P_diXPyVa2u4dMKsJsK03jIq0iS58DfpUk_Axh_7.

[13] Kemper J., Linde H. Challenges of passive infrared indoor localization. Proceedings of the 2008 5th Workshop on Positioning, Navigation and Communication.

[14] Arbula D., Ljubic S. (2020). Indoor localization based on infrared angle of arrival sensor network. Sensors.

[15] Yang C., Shao H.-R. (2015). WiFi-based indoor positioning. IEEE Commun. Mag..

[16] Zhang Y., Liu X., Wang Z., Chen H., Li M., Zhao J., Sun L., Yang Q., Xu T., Gao R. WiMap: Towards High-fidelity Wireless Signal Mapping for Enhanced Indoor Sensing. Proceedings of the 2024 ACM International Joint Conference on Pervasive and Ubiquitous Computing (UbiComp 2024).

[17] Singh A., Emam M., Al Mtawa Y. (2023). Comparative Analysis of Indoor Localization across Various Wireless Technologies. Eng.

[18] (2020). IEEE Standard for Information Technology—Telecommunications and Information Exchange Between Systems Local and Metropolitan Area Networks—Specific Requirements Part 11: Wireless LAN Medium Access Control (MAC) and Physical Layer (PHY) Specifications.

[19] Lim H., Kung L.-C., Hou J.C., Luo H. (2006). Proceedings of the IEEE INFOCOM 2006. 25TH IEEE International Conference on Computer Communications.

[20] Islam B., Islam M.T., Kaur J., Nirjon S. Lorain: Making a case for lora in indoor localization. Proceedings of the 2019 IEEE International Conference on Pervasive computing and Communications Workshops (PerCom Workshops).

[21] Wixted A.J., Kinnaird P., Larijani H., Tait A., Ahmadinia A., Strachan N. Evaluation of LoRa and LoRaWAN for wireless sensor networks. Proceedings of the 2016 IEEE SENSORS.

[22] Anjum M., Khan M.A., Hassan S.A., Mahmood A., Qureshi H.K., Gidlund M. (2020). RSSI fingerprinting-based localization using machine learning in LoRa networks. IEEE Internet Things Mag..

[23] Staniec K., Kowal M. (2018). LoRa performance under variable interference and heavy-multipath conditions. Wirel. Commun. Mob. Comput..

[24] Wang X., Gao L., Mao S., Pandey S. (2016). CSI-based fingerprinting for indoor localization: A deep learning approach. IEEE Trans. Veh. Technol..

[25] Ali I.T., Muis A., Sari R.F. (2021). A deep learning model implementation based on rssi fingerprinting for lora-based indoor localization. EUREKA Phys. Eng..

[26] Luo R.C., Hsiao T.J. (2018). Dynamic wireless indoor localization incorporating with an autonomous mobile robot based on an adaptive signal model fingerprinting approach. IEEE Trans. Ind. Electron..

[27] Khalajmehrabadi A., Gatsis N., Akopian D. (2017). Modern WLAN fingerprinting indoor positioning methods and deployment challenges. IEEE Commun. Surv. Tutor..

[28] Kjærgaard M.B. (2007). A taxonomy for radio location fingerprinting. International Symposium on Location-and Context-Awareness.

[29] Bahl P., Padmanabhan V.N. RADAR: An in-building RF-based user location and tracking system. Proceedings of the IEEE INFOCOM 2000. Conference on Computer Communications. Nineteenth Annual Joint Conference of the IEEE Computer and Communications Societies (Cat. No. 00CH37064).

[30] Youssef M., Agrawala A. The Horus WLAN location determination system. Proceedings of the 3rd International Conference on Mobile Systems, Applications, and Services.

[31] Madigan D., Einahrawy E., Martin R.P., Ju W.-H., Krishnan P., Krishnakumar A.S. (2005). Bayesian indoor positioning systems. IEEE Infocom.

[32] Farshad A., Li J., Marina M.K., Garcia F.J. A microscopic look at WiFi fingerprinting for indoor mobile phone localization in diverse environments. Proceedings of the International Conference on Indoor Positioning and Indoor Navigation.

[33] Han D., Jung S., Lee M., Yoon G. (2014). Building a practical Wi-Fi-based indoor navigation system. IEEE Pervasive Comput..

[34] He S., Chan S.-H.G. Sectjunction: Wi-Fi indoor localization based on junction of signal sectors. Proceedings of the 2014 IEEE International Conference on Communications (ICC).

[35] Feng C., Au W.S.A., Valaee S., Tan Z. (2011). Received-signal-strength-based indoor positioning using compressive sensing. IEEE Trans. Mob. Comput..

[36] Au A.W.S., Feng C., Valaee S., Reyes S., Sorour S., Markowitz S.N., Gold D., Gordon K., Eizenman M. (2012). Indoor tracking and navigation using received signal strength and compressive sensing on a mobile device. IEEE Trans. Mob. Comput..

[37] He S., Chan S.-H.G. (2015). Tilejunction: Mitigating signal noise for fingerprint-based indoor localization. IEEE Trans. Mob. Comput..

[38] Honkavirta V., Perala T., Ali-Loytty S., Piché R. A comparative survey of WLAN location fingerprinting methods. Proceedings of the 2009 6th Workshop on Positioning, Navigation and Communication.

[39] Nuno-Barrau G., Páez-Borrallo J.M. (2006). A new location estimation system for wireless networks based on linear discriminant functions and hidden Markov models. EURASIP J. Adv. Signal Process..

[40] Wu C.-L., Fu L.-C., Lian F.-L. WLAN location determination in e-home via support vector classification. Proceedings of the IEEE International Conference on Networking, Sensing and Control.

[41] Tang L., Zhang Z., Zhao Y., Feng T., Wong W.-C., Garg H.K. A comparison of WiFi-based indoor positioning methods. Proceedings of the 2019 13th International Conference on Signal Processing and Communication Systems (ICSPCS).

[42] He S., Chan S.-H.G. (2015). Wi-Fi fingerprint-based indoor positioning: Recent advances and comparisons. IEEE Commun. Surv. Tutor..

[43] Mirowski P., Milioris D., Whiting P., Ho T.K. (2014). Probabilistic radio-frequency fingerprinting and localization on the run. Bell Labs Tech. J..

[44] Redzic M., Brennan C., O’Connor N. (2013). SEAMLOC: Seamless Indoor Localisation Based on Reduced Number of Calibration Points. IEEE Trans. Mob. Comput..

[45] Roos T., Myllymäki P., Tirri H., Misikangas P., Sievänen J. (2002). A probabilistic approach to WLAN user location estimation. Int. J. Wirel. Inf. Netw..

[46] Jospin L.V., Laga H., Boussaid F., Buntine W., Bennamoun M. (2022). Hands-on Bayesian neural networks—A tutorial for deep learning users. IEEE Comput. Intell. Mag..

[47] Arigye W., Pu Q., Zhou M., Khalid W., Tahir M.J. (2022). RSSI Fingerprint Height Based Empirical Model Prediction for Smart Indoor Localization. Sensors.

[48] Pinkney J. (2003). Low Complexity Indoor Wireless Data Links Using Chirp Spread Spectrum. Ph.D. Thesis.

[49] Tsai Y.-R., Chang J.-F. The feasibility of combating multipath interference by chirp spread spectrum techniques over Rayleigh and Rician fading channels. Proceedings of the IEEE 3rd International Symposium on Spread Spectrum Techniques and Applications (ISSSTA’94).

[50] Kim K., Li S., Heydariaan M., Smaoui N., Gnawali O., Suh W., Suh M.J., Kim J.I. (2021). Feasibility of LoRa for smart home indoor localization. Appl. Sci..

[51] Vangelista L. (2017). Frequency shift chirp modulation: The LoRa modulation. IEEE Signal Process. Lett..

[52] Tong X., Wan Y., Li Q., Tian X., Wang X. (2021). CSI Fingerprinting Localization with Low Human Efforts. IEEE/ACM Trans. Netw..

[53] Golestanian M., Siva J., Poellabauer C., Ortiz J.H., de la Cruz A.P. (2017). Radio frequency-based indoor localization in ad-hoc networks. Ad Hoc Networks.

[54] Li H., Qian Z., Tian C., Wang X. (2020). TILoc: Improving the robustness and accuracy for fingerprint-based indoor localization. IEEE Internet Things J..

[55] Li M., Wang W. Hybrid Zone: Bridging Acoustic and Wi-Fi for Enhanced Gesture Recognition. Proceedings of the IEEE INFOCOM 2024—IEEE Conference on Computer Communications.

[56] Shahidul Islam M., Islam M.T., Almutairi A.F., Beng G.K., Misran N., Amin N. (2019). Monitoring of the human body signal through the Internet of Things (IoT) based LoRa wireless network system. Appl. Sci..

[57] Zhu H., Li S., Zheng L., Yang L. (2017). Modeling and validation on path loss of WSN in pig breeding farm. Trans. Chin. Soc. Agric. Eng..

[58] Elkhodr M., Shahrestani S., Cheung H. (2016). Emerging wireless technologies in the internet of things: A comparative study. arXiv.

[59] Ali A.I., Partal S.Z., Kepke S., Partal H.P. ZigBee and LoRa based wireless sensors for smart environment and IoT applications. Proceedings of the 2019 1st Global Power, Energy and Communication Conference (GPECOM).

[60] Haxhibeqiri J., Karaagac A., Van den Abeele F., Joseph W., Moerman I., Hoebeke J. LoRa indoor coverage and performance in an industrial environment: Case study. Proceedings of the 2017 22nd IEEE International Conference on Emerging Technologies and Factory Automation (ETFA).

[61] Kulkarni P., Hakim Q.O.A., Lakas A. (2019). Experimental evaluation of a campus-deployed IoT network using LoRa. IEEE Sens. J..

[62] Pietrosemoli E. (2017). Wireless Standards for IoT: WiFi, BLE, Sigfox, NB-IoT and LoRa.

[63] Petäjäjärvi J., Mikhaylov K., Pettissalo M., Janhunen J., Iinatti J. (2017). Performance of a low-power wide-area network based on LoRa technology: Doppler robustness, scalability, and coverage. Int. J. Distrib. Sens. Netw..

[64] Bornholdt L., Kaven S., Skwarek V. Adaptive procedure for indoor localization using LoRa devices. Proceedings of the 2021 International Conference on Indoor Positioning and Indoor Navigation (IPIN).

[65] Koledoye M.A., De Martini D., Rigoni S., Facchinetti T. A comparison of RSSI filtering techniques for range-based localization. Proceedings of the 2018 IEEE 23rd International Conference on Emerging Technologies and Factory Automation (ETFA).

[66] Goldsmith A. (2005). Wireless Communications.

[67] Andersen J.B., Rappaport T.S., Yoshida S. (1995). Propagation measurements and models for wireless communications channels. IEEE Commun. Mag..

[68] Nessa A., Adhikari B., Hussain F., Fernando X.N. (2020). A survey of machine learning for indoor positioning. IEEE Access.

[69] Lawrence N., Hyvärinen A. (2005). Probabilistic non-linear principal component analysis with Gaussian process latent variable models. J. Mach. Learn. Res..

[70] Alhomayani F., Mahoor M.H. (2020). Deep learning methods for fingerprint-based indoor positioning: A review. J. Locat. Based Serv..

[71] Wang Z., Bovik A.C., Sheikh H.R., Simoncelli E.P. (2004). Image quality assessment: From error visibility to structural similarity. IEEE Trans. Image Process..

[72] He T., Huang C., Blum B.M., Stankovic J.A., Abdelzaher T. Range-Free Localization Schemes for Large Scale Sensor Networks. Proceedings of the 9th annual International Conference on Mobile Computing and Networking.

[73] Patwari N., Ash J.N., Kyperountas S., Hero A.O., Moses R.L., Correal N.S. (2005). Locating the nodes: Cooperative localization in wireless sensor networks. IEEE Signal Process. Mag..

[74] Alsheikh M.A., Lin S., Niyato D., Tan H.P. (2014). Machine Learning in Wireless Sensor Networks: Algorithms and Applications. IEEE Commun. Surv. Tutor..

[75] Shi Y., Shi W., Liu X., Xiao X. (2020). An RSSI Classification and Tracing Algorithm to Improve Trilateration-Based Positioning. Sensors.

[76] Lutakamale A.S., Myburgh H.C., De Freitas A. (2023). RSSI-based Fingerprint Localization in LoRaWAN Networks Using PSO-Optimized Random Forest. Comput. Commun..

[77] Islam K.Z., Murray D., Diepeveen D., Jones M.G., Sohel F. (2021). Machine Learning-based LoRa Localisation Using Multiple Gateways. IET Wirel. Sens. Syst..

